# Low blood carotenoid status in dementia and mild cognitive impairment: A systematic review and meta-analysis

**DOI:** 10.1186/s12877-023-03900-7

**Published:** 2023-03-30

**Authors:** Lin Wang, Tie Zhao, Xu Zhu, Qinghua Jiang

**Affiliations:** 1 School of Pharmacy, China Medical University, Shenyang, China; 2 Department of Pharmacy, Shengjing Hospital of China Medical University, Shenyang, China

**Keywords:** Blood carotenoid levels, Dementia, Mild cognitive impairment, Meta-analysis, Systematic review

## Abstract

**Background:**

Given their potent antioxidation properties, carotenoids play a role in delaying and preventing dementia and mild cognitive impairment (MCI). However, observational studies have found inconsistent results regarding the associations between blood carotenoid levels and the risk of dementia and MCI. We conducted this systematic review and meta-analysis to investigate the relationship between blood carotenoid levels and the risk of dementia and MCI.

**Methods:**

A systematic search was performed in the Web of Science, PubMed, Embase, and Cochrane Library electronic databases to retrieve relevant English articles published from their inception until February 23, 2023. Study quality was assessed by the Newcastle-Ottawa scale. Standardized mean differences (SMDs) and 95% confidence intervals (CIs) were pooled using random-effect meta-analyses. Ultimately, 23 studies (n = 6610) involving 1422 patients with dementia, 435 patients with MCI, and 4753 controls were included.

**Results:**

Our meta-analysis showed that patients with dementia had lower blood lycopene (SMD: -0.521; 95%CI: -0.741, -0.301), *α*-carotene (SMD: -0.489; 95%CI: -0.697, -0.281), *β*-carotene (SMD: -0.476; 95%CI: -0.784, -0.168), lutein (SMD: -0.516; 95%CI: -0.753, -0.279), zeaxanthin (SMD: -0.571; 95%CI: -0.910, -0.232) and *β*-cryptoxanthin (SMD: -0.617; 95%CI: -0.953, -0.281) than the controls. Our results indicated that blood carotenoid levels were significantly lower in patients with dementia than in controls, despite high heterogeneity across the studies. Owing to insufficient data, we did not observe a similar and stable relationship between blood carotenoid levels and MCI.

**Conclusions:**

Our meta-analysis indicated that lower blood carotenoid levels may be a risk factor for dementia and MCI.

**Supplementary Information:**

The online version contains supplementary material available at 10.1186/s12877-023-03900-7.

## Background

Dementia is characterized by a progressive decline in cognitive performance and executive and social functioning [1]. Based on its clinical characteristics and etiologies, dementia is divided primarily into vascular dementia (VaD) and neurodegenerative dementia, including Alzheimer’s disease (AD) and Lewy-body dementia (LBD) [2]. Mild cognitive impairment (MCI) represents the prodromal stage of dementia; over time, the condition ultimately progresses to dementia [3]. Other than four cholinesterase inhibitors, memantine, and etiological strategies for delaying the course of dementia, there are currently few effective disease-modifying dementia treatments available [4]. Naturally, these are not sufficient to treat dementia after the manifestation of clinical symptoms. Therefore, the goal of dementia therapy should be changed from passive symptomatic treatment to proactive disease prevention. Several pathological studies have suggested that irreversible neuronal apoptosis or neuronal loss induced by oxidative stress plays an important role in the different types and degrees of dementia [5]. Therefore, early detection of antioxidant levels and reduction of oxidative stress can provide opportunities to avoid the deterioration in cognitive function and achieve satisfactory preventive efficacy.

Carotenoids are lipid-soluble conjugated polyene pigments that are primarily present in red and orange fruits and vegetables, such as carrots, tomatoes, watermelons, and guava [6]. Based on their molecular structures (presence or absence of oxygen atoms), carotenoids can be classified into two major categories: carotenes (C_40_H_56_) and hydroxy-substituted carotenes, xanthophylls (C_40_H_56_O_2_) [7]. In humans, the former mainly includes lycopene, *α-*, and *β*-carotenes, while the latter mainly includes lutein, zeaxanthin, canthaxanthin, and *β*-cryptoxanthin [8, 9]. Lipophilic structures of carotenoids can allow them to cross the blood-brain barrier (BBB) and accumulate in the brain [10]. The existence of polyene chains in carotenoids determines their chemical properties to scavenge free radicals [11]. Hence, carotenoids exhibit excellent antioxidative and neuroprotective properties and have garnered significant attention in preventing and treating neurological diseases. Several animal studies have shown that oral carotenoid treatments attenuated oxidative stress and cognitive impairment in AD and VaD models [12–17]. Such carotenoid intervention tended to be protective against neurodegenerative processes including a cognitive decline in epidemiologic trials [18–20]. It should be pointed out that blood carotenoids in humans are mainly got from diet intake or antioxidant supplements [21]. So, after carotenoid ingestion, can the pathological or physiological status of dementia/MCI and healthy populations affect the blood carotenoid levels?

A multitude of trial studies have reported that dementia or cognitive decline was significantly linked to different plasma carotenoid levels, but not consistently [22, 23]. In addition, a recent study meta-analysis explored the association between plasma/serum carotenoids and AD and found that only plasma/serum lutein and zeaxanthin levels were associated with a reduced risk of AD [24]. However, this study did not detect the relevance between other carotenoids and AD risk on one hand and did not extensively examine the associations of blood carotenoids with the risk of MCI and other dementia subtypes, such as VaD and LBD. In contrast, another study found that higher plasma *trans*-*β*‐carotene and α-carotene tended to be associated with a higher risk of AD [25]. Thus, given the importance of disease prevention and the incompleteness of previous research, we aimed to perform a more comprehensive and systematic review and meta-analysis to summarize data from previous studies and clarify the association between blood carotenoid levels and multiple dementia subtypes and MCI.

## Materials and Methods

### Study design

This systematic review and meta-analysis adhered to the Preferred Reporting Items for Systematic Reviews and Meta-Analyses [26]. The review protocol was registered prior to conducting the study and is published online in the PROSPERO database of systematic reviews (https://www.crd.york.ac.uk/prospero/registration number # CRD42020176174).

### Patient and public involvement statement

This meta-analysis was based on data obtained from several databases. Patients and the public were not directly involved in this study.

### Search strategy

A systematic and comprehensive search was performed using the Web of Science, PubMed, Embase, and Cochrane Library electronic databases, from their inception until February 23, 2023. Relevant publications were initially searched using multiple keywords as follows: “carotenoids”, “lycopene”, “carotene”, “lutein”, “zeaxanthin”, “cryptoxanthin”, “plasma”, “serum”, “dementia”, “vascular dementia”, “Lewy bodies dementia”, “Alzheimer’s disease”, “mild cognitive impairment”, and “MCI”, following controlled vocabulary terms “AND” and “OR”. The relevance of the retrieved papers was evaluated by reviewing their titles and abstracts. The search was limited to peer-reviewed articles published in English. Duplicates of studies were identified and excluded. We also manually screened the reference lists of the relevant articles and reviewed the relevant studies.

### Inclusion and exclusion criteria

Two investigators independently evaluated the studies identified for analysis. Any disagreements involving inclusion or exclusion were resolved by a third investigator (QHJ) through consensus and adjudication. In this meta-analysis, eligible studies were selected if they met the following inclusion criteria: (1) case-control, cross-sectional and case-cohort studies with dementia/MCI and control groups; (2) diagnosis of MCI and dementia based on NINDS-AIREN or NINCDS-ADRDA or other diagnostic criteria; (3) articles published in English; and (4) reported plasma or serum carotenoid concentrations. Studies were excluded if they met any of the following exclusion criteria: (1) reviews, letters, book chapters, short communications, abstracts, editorials, or case reports; (2) studies without normal cognition controls; (3) studies without available detailed data; (4) animal or in vitro experiments; (5) duplicated publications; (6) studies of patients without dementia or cognitive examinations and (7) dead patients.

### Data extraction

Relevant key information, including first author names, publication year, country, diagnostic criteria, analytical methods, sample sources, dementia type, participant characteristics, and blood carotenoid concentrations, were independently extracted from each selected study by two investigators and re-examined for accuracy by the third investigator. In included studies that reported median and range data, these values were converted into means and standard deviations (SD) using the equations of Hozo et al. [27].

### Quality assessment

The overall study quality was independently assessed and scored by two investigators using the Newcastle-Ottawa Scale (NOS) [28], which was based on the following three quality parameters: (1) selection (case definition, representativeness, control selection, control definition; 4 points), (2) comparability (comparability of cases and controls based on the design or analysis; 2 points), and (3) outcome (ascertainment of exposure, the same method of ascertainment for cases and controls, and nonresponse rate; 3 points). The total points varied from 0 to 9; studies with ≥ 7 points were generally considered high-quality; studies that ranged between 5 and 6 points were considered moderate quality, and studies with points < 5 were considered low-quality. The details of these criteria and points of individual studies are listed in Supplemental Table 1.

### Data synthesis and statistical analysis

All statistical analyses were performed using STATA 15.0 software (Stata Corp, College Station, TX, USA). Standardized mean differences (SMDs) and 95% confidence intervals (CIs) were considered measures of continuous outcomes in all studies. Statistical heterogeneity across the studies was assessed using the *I*^*2*^ statistic. *I*^2^ values < 50% indicated low heterogeneity, and in such cases, a fixed-effect model was selected; if *I*^2^ values were > 50%, which indicated high heterogeneity, a random-effect model was employed [29]. Subgroup analyses for disease type and sample source were conducted to ascertain the potential sources of heterogeneity. If enough studies were available (≥ 10 studies), meta-regression was performed on mean age, country, percentage of men, NOS score, and diagnostic criteria as numerical covariates to further investigate the association between blood carotenoid levels and disease with dementia and MCI [30]. Sensitivity analyses were performed by excluding one study at a time to assess the robustness of the pooled SMD [31]. Funnel plots and Egger’s test were used to analyze possible publication bias when the number of studies permitted (≥ 10 studies) [32]. For all analyses, a two-tailed *P* value < 0.05 was considered statistically significant.

## Results

### Search results

**Fig. 1:**
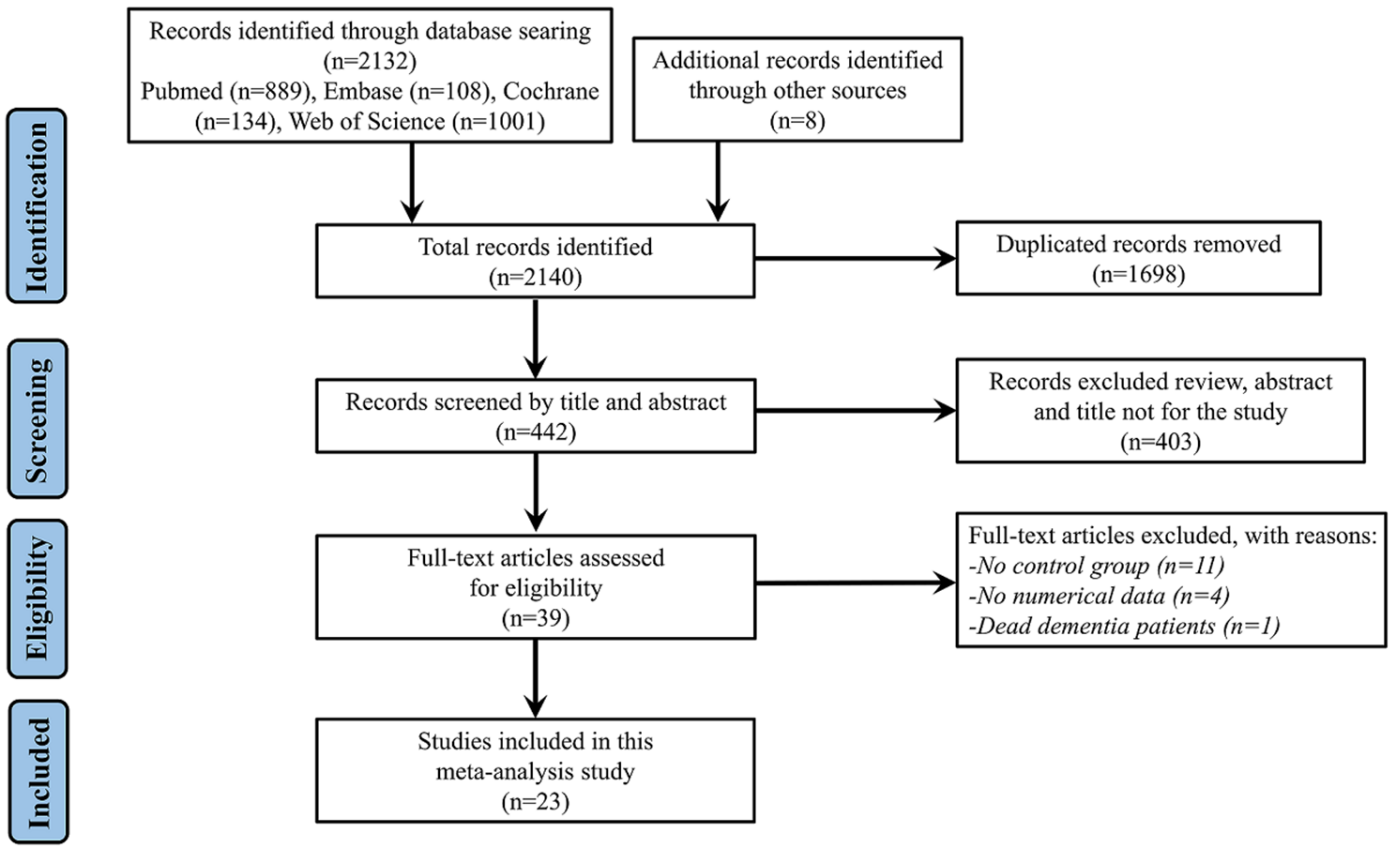
Flowchart of study selection process

A flow chart of the study search and selection process is presented in Fig. 1. Altogether, 2140 articles were obtained from the electronic databases in the initial search, and 1698 duplicate files were deleted. After careful screening of titles and abstracts, 403 unrelated articles, such as reviews, abstracts, or animal studies, were removed. Consequently, 23 studies were eligible for inclusion in this systematic review and meta-analysis.

### General characteristics

The total number of participants was 6610 (50.1–85.9 years), including 1422 with dementia, 435 with MCI, and 4753 controls. Of these, 19 and 4 studies were used for meta-analyses investigating the association between blood carotenoid levels and dementia and MCI, respectively. All the trials were performed between 1998 and 2020, and 13 were published after 2010. These studies included data on lycopene (n = 16), *α*-carotene (n = 13), *β*-carotene (n = 17), lutein (n = 11), zeaxanthin (n = 11), and *β*-cryptoxanthin (n = 9) levels. Fifteen studies were conducted in Europe and two in the USA, with only one in South America, one in Oceania, and one in Asia. The blood carotenoid levels in included studies were measured using high-performance liquid chromatography. The sample size varied from 10 to 1628. The proportion of men patients varied from 0 to 71%. The quality scores of the included studies ranged from 6 to 9, with a mean score of 7.8. The majority of the studies were of high quality, and three studies were of moderate quality. The detailed characteristics of each included study are provided in Table 1.

**Table 1:** General characteristics of the included studies

Study Author(reference)	Country	Diagnostic criteria	Analytical methods	Sample sources*	Patients’ MMSE at diagnosis	Type of dementia	Sample size		Gender (%male)		Mean age (years, mean ± SD)		Carotenoids levels (µmol/L, mean ± SD)		
							Patients	Controls	Patients	Controls	Patients	Controls	Carotenoids	Patients	Controls
Sinclair et al. [52]	UK	NINCDS-ADRDA; NINDS-AIREN	HPLC	Plasma	17.3 ± 4.3	AD	25	41	60	42	74.3 ± 8.1	73.4 ± 7.2	*β*-carotene	0.52 ± 0.32	0.52 ± 0.35
					17.8 ± 5.8	VaD	17	41	71	42	75.5 ± 6.3	73.4 ± 7.2	*β*-carotene	0.75 ± 0.46	0.52 ± 0.35
Foy et al. [33]	UK	DSM IV; NINCDS-ADRDA; NINDS-AIREN	HPLC	Plasma	> 10, ≤ 24	AD	79	58	62	55	79.0 ± 1.8	74.0 ± 2.8	lycopene	0.09 ± 0.04	0.09 ± 0.06
													*α*-carotene	0.04 ± 0.01	0.04 ± 0.01
													*β*-carotene	0.31 ± 0.04	0.31 ± 0.09
						VaD	37	58	51	55	79.0 ± 4.0	74.0 ± 2.8	lycopene	0.08 ± 0.03	0.09 ± 0.06
													*α*-carotene	0.03 ± 0.01	0.04 ± 0.01
													*β*-carotene	0.18 ± 0.05	0.31 ± 0.09
Jimenez et al. [47]	Spain	AD/STAD	HPLC	Serum	12.9 ± 5.8	AD	38	42	34	48	71.8 ± 7.0	70.1 ± 8.5	*α*-carotene	0.06 ± 0.06	0.07 ± 0.06
													*β*-carotene	0.21 ± 0.14	0.32 ± 0.26
Schippling et al. [50]	Germany	NINCDS-ADRDA; DSM-IV	HPLC	Plasma	19.0 ± 5.0	AD	29	29	48	45	71.7 ± 10.1	55.1 ± 18.8	*α*-carotene	0.06 ± 0.07	0.18 ± 0.06
													*β*-carotene	0.54 ± 0.51	0.56 ± 0.34
Mecocci et al. [34]	Italy	NINCDS-ADRDA; DSM-IV	HPLC	Plasma	17.3 ± 2.1	AD	40	39	50	49	75.9 ± 5.4	74.8 ± 6.3	lycopene	0.29 ± 0.13	0.72 ± 0.26
													*α*-carotene	0.04 ± 0.02	0.06 ± 0.03
													*β*-carotene	0.18 ± 0.06	0.52 ± 0.23
													lutein	0.35 ± 0.18	0.38 ± 0.17
													zeaxanthin	0.06 ± 0.03	0.11 ± 0.04
													*β*-cryptoxanthin	0.03 ± 0.02	0.30 ± 0.19
Polidori and Mecocci [35]	France	NINCDS-ADRDA	HPLC	Plasma	-	AD	35	40	0	0	85.9 ± 5.5	85.4 ± 4.4	lycopene	0.38 ± 0.09	0.72 ± 0.19
													*α*-carotene	0.04 ± 0.01	0.07 ± 0.07
													*β*-carotene	0.21 ± 0.11	0.24 ± 0.11
													lutein	0.33 ± 0.16	0.55 ± 0.21
													zeaxanthin	0.06 ± 0.06	0.07 ± 0.08
													*β*-cryptoxanthin	0.21 ± 0.11	0.26 ± 0.16
Quinn et al. [36]	USA	NINCDS-ADRDA	HPLC	Plasma	19.3 ± 7.0	AD	10	10	60	60	65.0 ± 7.0	65.0 ± 6.0	lycopene	0.44 ± 0.17	0.39 ± 0.19
													*α*-carotene	0.10 ± 0.16	0.15 ± 0.28
													*β*-carotene	0.36 ± 0.44	0.83 ± 0.98
Rinaldi et al. [37]	Italy	NINCDS-ADRDA; Clock Drawing test	HPLC	Plasma	26.9 ± 2.0	MCI	25	56	44	36	75.8 ± 4.8	75.8 ± 7.2	lycopene	0.84 ± 0.38	0.79 ± 0.36
													*α*-carotene	0.06 ± 0.07	0.13 ± 0.12
													*β*-carotene	0.56 ± 0.29	0.57 ± 0.33
													lutein	0.51 ± 0.18	0.69 ± 0.34
													zeaxanthin	0.11 ± 0.04	0.16 ± 0.08
													*β*-cryptoxanthin	0.45 ± 0.36	0.43 ± 0.42
					13.5 ± 6.5	AD	63	56	27	36	76.8 ± 6.9	75.8 ± 7.2	lycopene	0.62 ± 0.23	0.79 ± 0.36
													*α*-carotene	0.07 ± 0.03	0.13 ± 0.12
													*β*-carotene	0.59 ± 0.28	0.57 ± 0.33
													lutein	0.37 ± 0.19	0.69 ± 0.34
													zeaxanthin	0.06 ± 0.03	0.16 ± 0.08
													*β*-cryptoxanthin	0.17 ± 0.14	0.43 ± 0.42
Polidori et al. [38]	Germany	NINDS-AIREN; NINCDS-ADRDA	HPLC	Plasma	20.4 ± 3.0	AD	63	55	27	35	76.8 ± 6.9)	75.7 ± 7.3	lycopene	0.61 ± 0.22	0.78 ± 0.36
													*α*-carotene	0.06 ± 0.03	0.12 ± 0.12
													*β*-carotene	0.57 ± 0.28	0.55 ± 0.34
													lutein	0.36 ± 0.18	0.67 ± 0.35
													zeaxanthin	0.06 ± 0.02	0.15 ± 0.08
													*β*-cryptoxanthin	0.17 ± 0.14	0.41 ± 0.42
					19.8 ± 3.0	VaD	23	55	39	35	78.0 ± 6.5)	75.7 ± 7.3	lycopene	0.61 ± 0.22	0.78 ± 0.36
													*α*-carotene	0.06 ± 0.03	0.12 ± 0.12
													*β*-carotene	0.53 ± 0.27	0.55 ± 0.34
													lutein	0.34 ± 0.10	0.67 ± 0.35
													zeaxanthin	0.07 ± 0.05	0.15 ± 0.08
													*β*-cryptoxanthin	0.17 ± 0.12	0.41 ± 0.42
Wang et al. [39]	USA	NINCDS-ADRDA	HPLC	Plasma	21.9 ± 3.1	AD	36	10	44	50	75.6 ± 9.7	70.2 ± 11.2	lycopene	0.59 ± 0.34	0.55 ± 0.28
													*α*-carotene	0.13 ± 0.12	0.13 ± 0.12
													*β*-carotene	0.54 ± 0.44	0.66 ± 0.51
													lutein	0.26 ± 0.14	0.25 ± 0.14
													zeaxanthin	0.05 ± 0.03	0.04 ± 0.02
													*β*-cryptoxanthin	0.19 ± 0.16	0.15 ± 0.13
von Arnim et al. [40]	Germany	MMSE	HPLC	Serum	22.6 ± 1.9	ID	74	148	67	68	81.0 ± 2.3	79.0 ± 2.0	lycopene	0.21 ± 0.03	0.25 ± 0.05
													*β*-carotene	0.31 ± 0.04	0.46 ± 0.08
Giavarotti et al. [41]	Brazil	MMSE; CDR	HPLC	Plasma	< 24	AD	23	42	-	-	-	-	lycopene	0.67 ± 0.13	0.73 ± 0.09
													*β*-carotene	0.76 ± 1.00	0.79 ± 0.90
Nolan et al. [54]	Italy	MMSE; Clock drawing test; Semantic fluency score	HPLC	Serum	18.8 ± 3.7	AD	36	33	36	52	80.0 ± 7.8	76.0 ± 6.6	lutein	0.22 ± 0.12	0.30 ± 0.18
													zeaxanthin	0.05 ± 0.03	0.07 ± 0.04
Nolan et al. [55]	Italy	MMSE	HPLC	Serum	19.0 ± 3.7	AD	31	31	42	58	80.0 ± 7.8	76.0 ± 6.6	lutein	0.23 ± 0.11	0.30 ± 0.18
													zeaxanthin	0.05 ± 0.04	0.07 ± 0.04
Feart et al. [42]	France	MMSE; Isaac’s set test; Benton visual retention test	HPLC	Plasma	-	ID	119	893	-	-	-	-	lycopene	0.42 ± 0.29	0.45 ± 0.31
													*α*-carotene	0.18 ± 0.15	0.18 ± 0.16
													*β*-carotene	0.68 ± 0.51	0.74 ± 0.59
													lutein	0.27 ± 0.13	0.29 ± 0.15
													zeaxanthin	0.07 ± 0.04	0.07 ± 0.04
													*β*-cryptoxanthin	0.28 ± 0.21	0.30 ± 0.24
Amadieu et al. [48]	France	DSM-IV; NINCDS-ADRDA	HPLC	Plasma	-	ID	110	556	33	40	75.9 ± 4.2	72.8 ± 4.4	lycopene	0.43 ± 0.28	0.51 ± 0.32
													*α*-carotene	0.16 ± 0.12	0.18 ± 0.15
													*β*-carotene	0.63 ± 0.42	0.76 ± 0.57
													lutein	0.29 ± 0.15	0.30 ± 0.15
													zeaxanthin	0.07 ± 0.05	0.07 ± 0.04
													*β*-cryptoxanthin	0.27 ± 0.20	0.31 ± 0.23
Mullan et al. [43]	UK	NINCDS-ADRDA	HPLC	Serum	18.0 ± 6.5	AD	251	308	36	39	80.2 ± 7.7	76.5 ± 6.7	lycopene	0.32 ± 0.43	0.54 ± 0.60
													*α*-carotene	0.02 ± 0.02	0.03 ± 0.02
													*β*-carotene	0.10 ± 0.07	0.14 ± 0.11
													lutein	0.04 ± 0.03	0.05 ± 0.03
													zeaxanthin	0.01 ± 0.01	0.01 ± 0.01
													*β*-cryptoxanthin	0.01 ± 0.01	0.02 ± 0.01
Ayromlou et al. [49]	Iran	DSM-IV; GDS	HPLC	Serum	-	MCI	45	45	49	51	68.1 ± 5.3	69.6 ± 5.3	lycopene	0.03 ± 0.01	0.03 ± 0.01
													*β*-carotene	0.03 ± 0.01	0.04 ± 0.01
Boccardi et al. [44]	Italy	GDS, MMSE	HPLC	Plasma	20.1 ± 6.1	AD	31	37	19	43	79.3 ± 4.6	79.1 ± 5.1	lycopene	0.83 ± 0.17	0.93 ± 0.11
Rietman et al. [51]	Netherlands	Global cognitive functioning	HPLC	Plasma	-	MCI	199	1628	62	49	64.3 ± 8.5	53.4 ± 11.0	*α*-carotene	0.12 ± 0.02	0.15 ± 0.02
													*β*-carotene	0.48 ± 0.08	0.58 ± 0.09
													zeaxanthin	0.04 ± 0.01	0.04 ± 0.01
													*β*-cryptoxanthin	0.15 ± 0.05	0.22 ± 0.04
Dias et al. [45]	Germany	NINCDS-ADRDA	HPLC	Plasma	18.0 ± 7.0	VaD	44	33	39	33	80.1 ± 5.3	73.2 ± 8.3	lutein	0.27 ± 0.32	0.35 ± 0.41
													lycopene	0.32 ± 0.45	0.39 ± 0.61
													zeaxanthin	0.06 ± 0.07	0.08 ± 0.08
Mangialasche et al. [53]	Europe	NINCDS-ADRDA; DSM-IV	HPLC	Plasma	27.1 ± 1.8	MCI	166	187	42	46	75.8 ± 5.6	74.7 ± 5.3	*β*-carotene	0.47 ± 0.05	0.52 ± 0.04
					20.2 ± 4.7	AD	168	187	32	46	77.4 ± 6.3	74.7 ± 5.3	*β*-carotene	0.44 ± 0.06	0.52 ± 0.04
Sharma et al. [46]	Germany	MMSE	HPLC	Plasma	12–25	AD	40	35	50	40	72.9 ± 5.0	68.3 ± 10.4	*α*-carotene	0.14 ± 0.13	0.23 ± 0.27
													*β*-carotene	0.50 ± 0.41	0.56 ± 0.42
													*β*-cryptoxanthin	0.12 ± 0.11	0.19 ± 0.17
													lycopene	0.95 ± 0.69	0.95 ± 0.62

Abbreviation: AD, Alzheimer’s disease; CDR, clinical dementia rating; DSM, diagnostic and statistical manual of mental disorders; FACT-Cog, functional assessment of cancer therapy-cognitive function; GDS, geriatric depression scale; HPLC, high performance liquid chromatography; ICD, international Classification of diseases; ID, indefinite dementia; MCI, mild cognitive impairment; MID, multi-infarct dementia; MMSE, mini mental state examination; MoCA, montreal cognitive assessment; NIHTB-CB, neurological and behavioral function cognitive function battery; NINDS-AIREN, national institute of neurological disorders and stroke and association internationale pour la recherche et l’enseignementen neurosciences; NINCDS-ADRDA, national institutes of neurological and communicative disorders and stroke/Alzheimer’s disease and related disorders association; VaD, vascular dementia

*Blood samples were taken, and only all patients were diagnosed as probable dementia according to clinical, neurological, and/or neuropsychological evaluation

### Lycopene

**Fig. 2:**
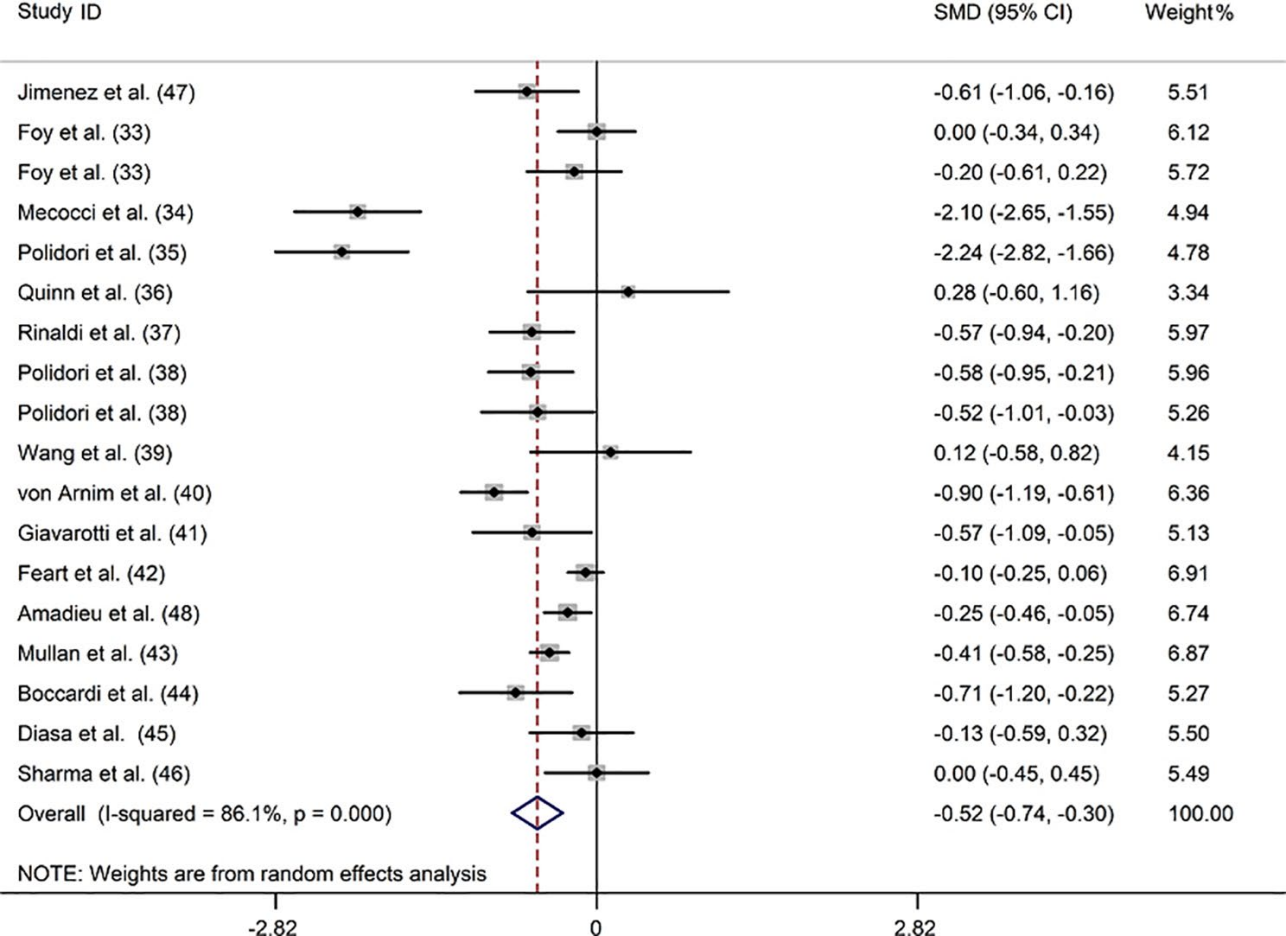
Forest plot of the relation between blood lycopene (µmol/L) and dementia and MCI, 1999–2020. The data are expressed as SMDs with 95%CIs. The overall effect is represented by a hollow diamond. The horizontal lines represent 95%CI. The sizes of the shaded squares are proportional to study weight

Sixteen studies [33–48] investigated blood lycopene concentrations in 1078 patients with dementia compared with 2433 controls. Dementia was positively associated with reduced blood lycopene levels in a random-effect model (SMD: -0.521; 95%CI: -0.741, -0.301) with a considerable degree of heterogeneity across the studies (*I*^2^ = 86.1%) (Fig. 2). Subgroup analysis accounting for dementia type revealed that this association was found for AD and VaD but not for indefinite dementia (ID). Additionally, subgroup analysis stratified by sample source showed that lycopene levels were reduced in both the plasma and serum of patients with dementia (Supplemental Table 2). Meta-regression analysis revealed that the mean age of patients, proportion of men, country, NOS score, and diagnostic criteria did not contribute to the heterogeneity of the included studies (Supplemental Table 3). The sensitivity analysis illustrated that removing any of the studies did not significantly affect the outcome (Supplemental Fig. 1A). Moreover, no publication bias was observed according to Egger’s test (*P* = 0.686) (Supplemental Table 4).

Two studies evaluated the blood lycopene levels in 70 patients with MCI compared to 101 controls [37, 49]. One study reported remarkably lower plasma levels of lycopene in patients with MCI than that in controls (*P* < 0.05) [49]. In the other study, no significant difference in serum lycopene levels was reported between the two groups [37]. Our investigation found no noticeable association between lower blood lycopene levels and MCI (SMD: 0.059; 95%CI: -0.252, 0.370), applying a fixed-effect model with low heterogeneity (*I*^*2*^ = 0.0%) (Supplemental Fig. 3).

### *α*-Carotene

**Fig. 3:**
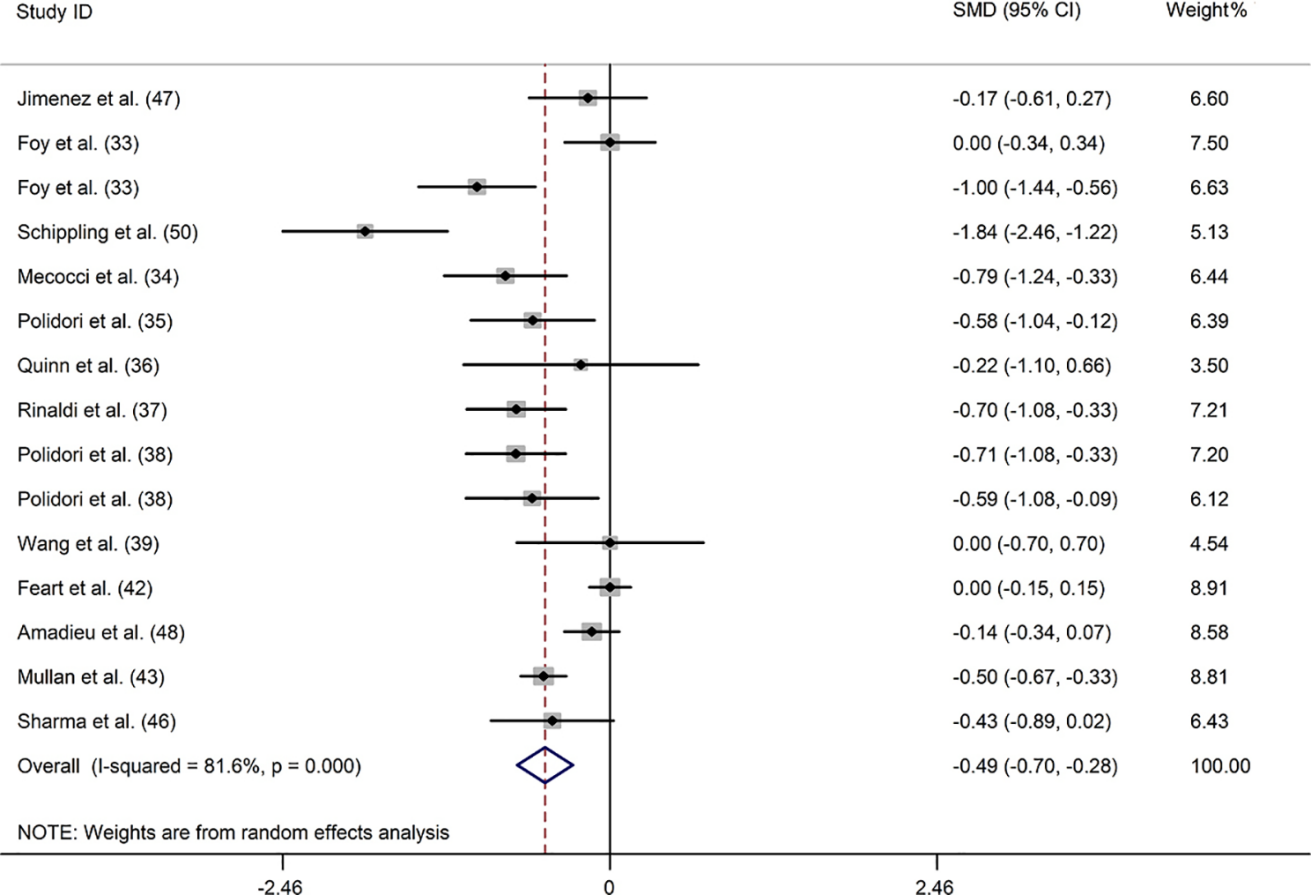
Forest plot of the relation between blood *α*-carotene (µmol/L) and dementia and MCI, 1999–2020. The data are expressed as SMDs with 95%CIs. The overall effect is represented by a hollow diamond. The horizontal lines represent 95%CI. The sizes of the shaded squares are proportional to study weight

Thirteen studies [33–39, 42, 43, 46–48, 50] compared blood *α*-carotene concentrations in 973 patients with dementia and 2244 controls. Meta-analysis results revealed that patients with dementia had remarkably lower blood *α*-carotene levels compared with the controls (SMD: -0.489; 95%CI: -0.697, -0.281). Because of the substantial heterogeneity across the studies (*I*^2^ = 81.6%), a random-effect model was employed (Fig. 3). Subgroup analysis indicated that reduced *α*-carotene levels were observed in patients with AD and VaD and both plasma and serum samples (Supplemental Table 2). Furthermore, meta-regression analysis showed that none of the aforementioned covariates had a marked impact on heterogeneity (Supplemental Table 3). Sensitivity analysis showed that the inverse association was not completely reversed by omitting any one study, which in turn suggested that the stability of the overall result was robust (Supplemental Fig. 1B). Moreover, a visual funnel plot revealed approximate symmetry (Supplemental Fig. 2A). Egger’s test showed no evidence of publication bias for blood *α*-carotene levels (*P* = 0.956) (Supplemental Table 4).

Two studies [37, 51] examining blood *α*-carotene levels in 224 patients with MCI and 1684 controls reported that patients with MCI exhibited a slight decrease in lycopene levels compared to controls (*P* < 0.05), which was in line with the pooled findings (SMD: -1.108; 95%CI: -1.937, -0.280). However, significant heterogeneity was observed in the pooled analysis (*I*^*2*^ = 90.7%) (Supplemental Fig. 3).

### *β*-Carotene

**Fig. 4:**
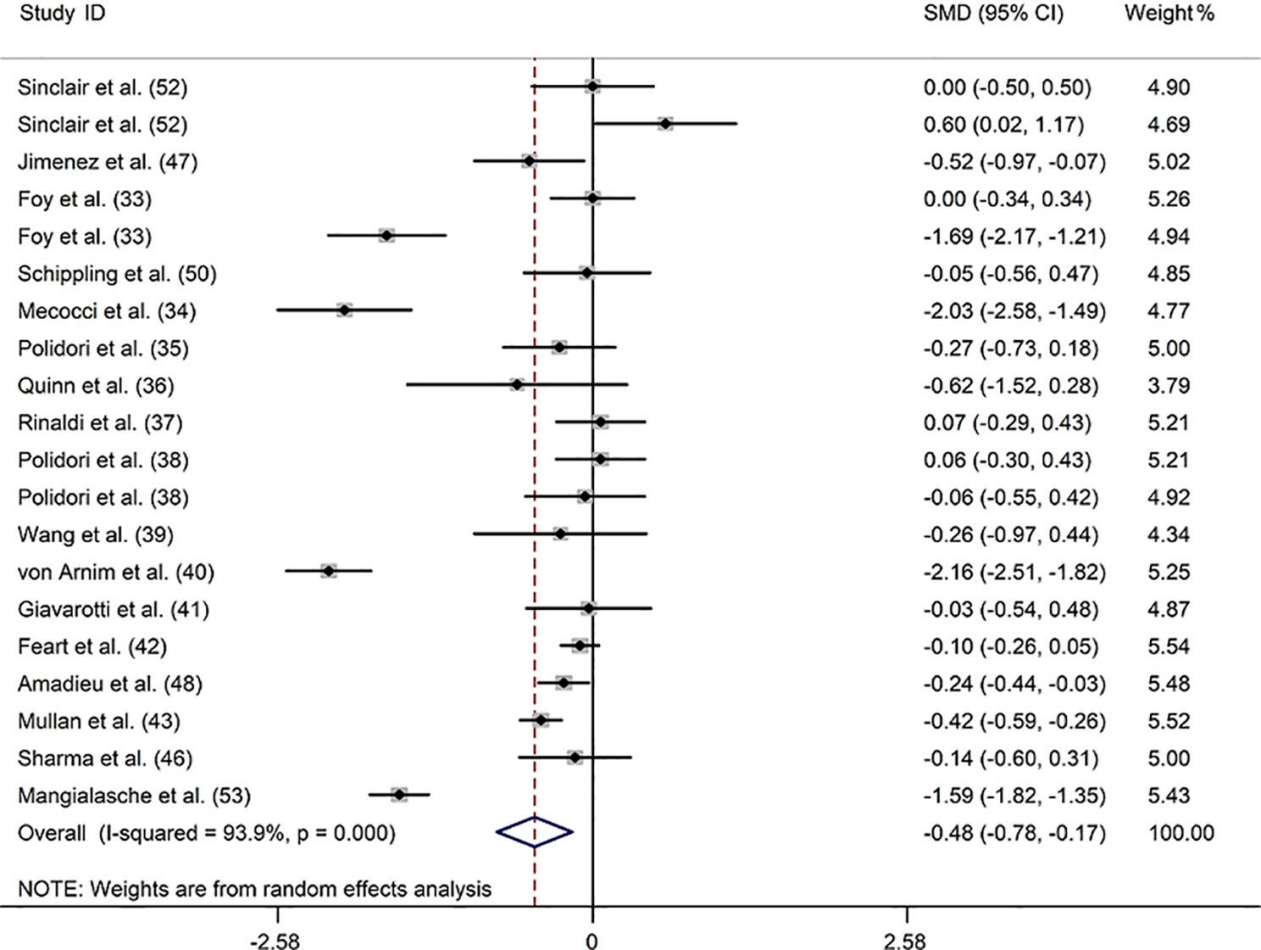
Forest plot of the relation between blood *β*-carotene (µmol/L) and dementia and MCI, 1998–2020. The data are expressed as SMDs with 95%CIs. The overall effect is represented by a hollow diamond. The horizontal lines represent 95%CI. The sizes of the shaded squares are proportional to study weight

Seventeen articles [33–43, 46–48, 50, 52, 53] including 1280 patients with dementia and 2703 controls provided relevant data regarding the link between blood *β*-carotene levels and dementia. There was an association between low blood *β*-carotene levels and dementia in a random-effect model (SMD: -0. 476; 95%CI: -0.784, -0.168) with substantial heterogeneity among these trials (*I*^*2*^ = 93.9%) (Fig. 4). Subgroup analysis found no significant difference in *β*-carotene concentrations between patients with dementia and controls when the dementia type was not restricted to AD or the sample source was serum (Supplemental Table 2). The meta-regression analysis showed that none of the above-mentioned covariates owned an impact on heterogeneity. In addition, none of the available moderators could explain the source of the heterogeneity (Supplemental Table 3). Based on sensitivity analysis, the final SMD did not change appreciably after each study was excluded, indicating a stable meta-analysis result (Supplemental Fig. 1C). Egger’s test detected no publication bias in any of the included studies (*P* = 0.284) (Supplemental Table 4).

Four studies [37, 49, 51, 53] investigating the link between blood *β*-carotene levels and MCI were included, including 435 patients with MCI and 1916 controls. Of these studies, two studies [37, 49] reported that blood *β*-carotene levels did not differ between patients with MCI and controls; however, two studies [51, 53] reported markedly lower plasma *β*-carotene levels in patients with MCI than in controls. Pooled analysis showed a negative correlation between blood *β*-carotene levels and MCI (SMD: -0.872; 95%CI: -1.226, -0.518; *I*^*2*^ = 84.4%) (Supplemental Fig. 3).

### Lutein

**Fig. 5:**
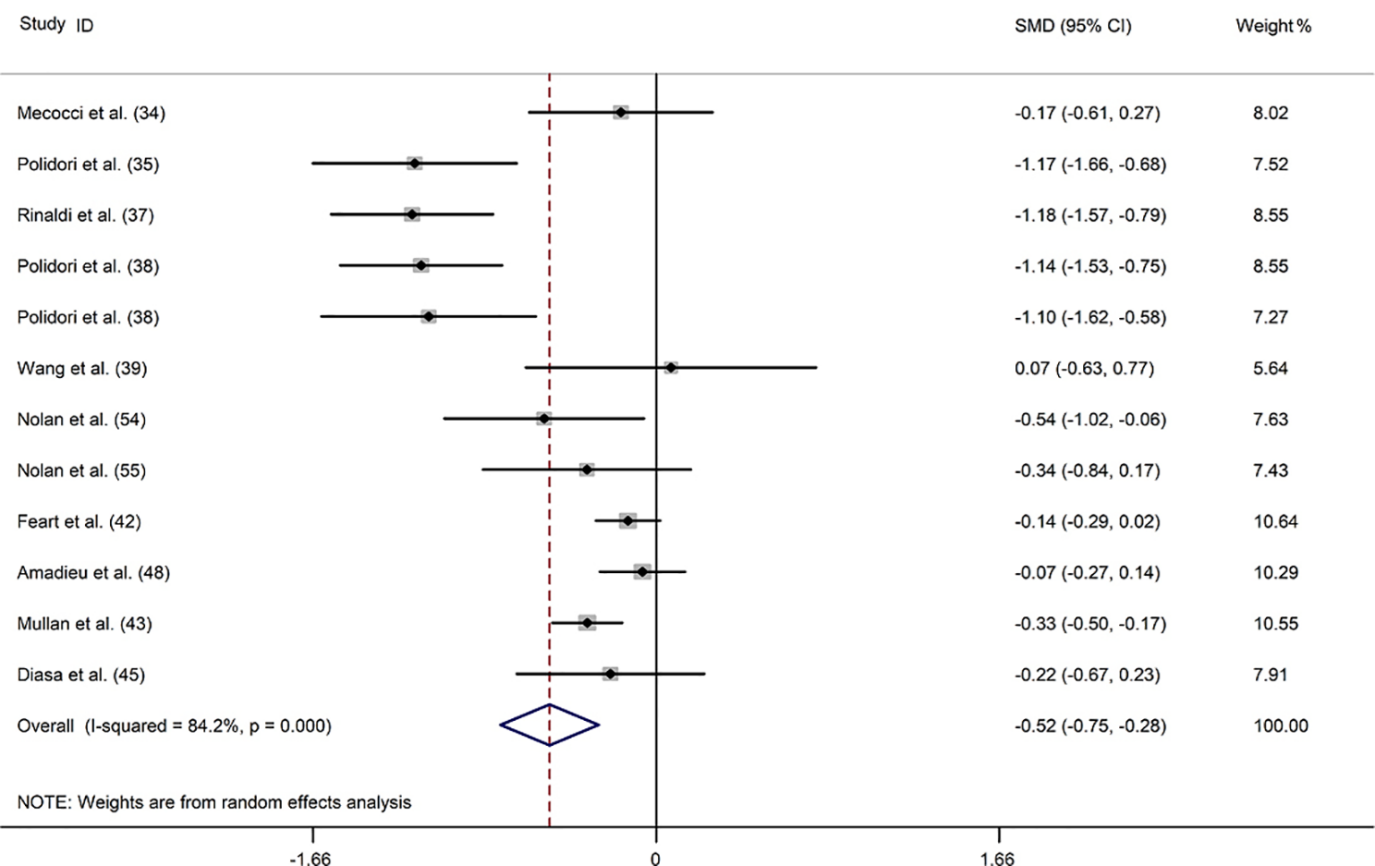
Forest plot of the relation between blood lutein (µmol/L) and dementia and MCI, 2002–2017. The data are expressed as SMDs with 95%CIs. The overall effect is represented by a hollow diamond. The horizontal lines represent 95%CI. The sizes of the shaded squares are proportional to study weight

Eleven studies [16, 34, 35, 37–39, 42, 43, 45, 48, 54] reported blood lutein levels in 851 patients with dementia and 2109 controls. Meta-analysis results showed that patients with dementia had lower blood lutein levels compared with the controls (SMD: -0.516; 95%CI: -0.753, -0.279), and the heterogeneity was significant (*I*^*2*^ = 84.2%) (Fig. 5). In the subgroup analysis by dementia type, lower blood lutein levels were observed in patients with AD and VaD but not in patients with ID. Concerning the sample source, a significant relationship was noted in studies assessing lutein levels in both plasma and serum samples (Supplemental Table 3). In the meta-regression analysis, the percentages of male and NOS score led to 40.63% (*P* = 0.036) and 20.84% (*P* = 0.119) heterogeneity, respectively (Supplemental Table 3). Moreover, the sensitivity analysis showed that the statistical significance of the meta-analysis results was not significantly altered after omitting any of these studies (Supplemental Fig. 1D). The funnel plot appeared approximately symmetric, and no publication bias was found according to Egger’s test (*P* = 0.959) (Supplemental Fig. 2B and Table 4).

A single publication [37] that investigated plasma lutein levels in 25 patients with MCI and 56 controls reported significantly low plasma lutein concentrations in patients with MCI (*P* < 0.01) (Supplemental Fig. 3).

### Zeaxanthin

**Fig. 6:**
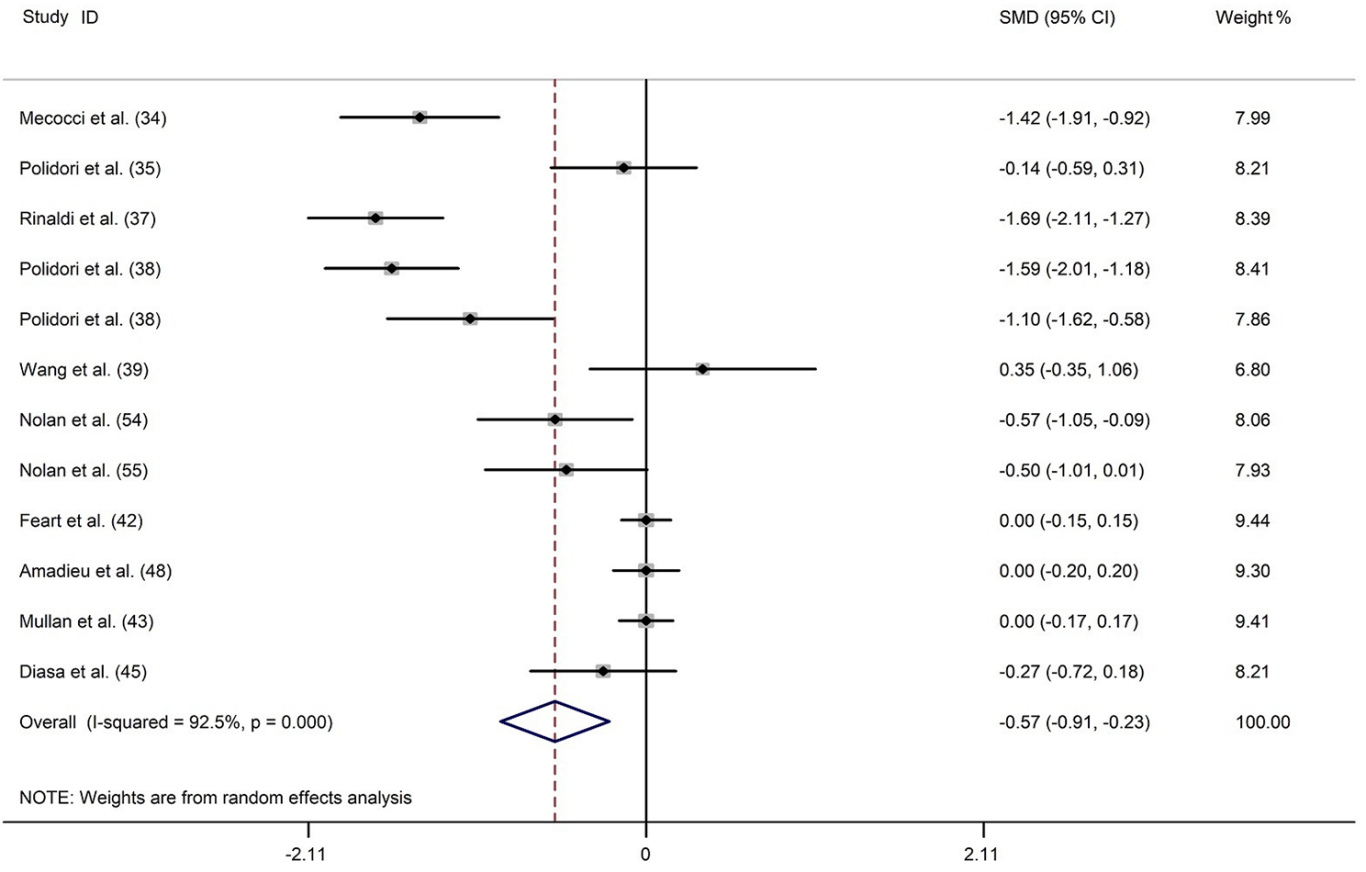
Forest plot of the relation between blood zeaxanthin (µmol/L) and dementia and MCI, 2002–2017. The data are expressed as SMDs with 95%CIs. The overall effect is represented by a hollow diamond. The horizontal lines represent 95%CI. The sizes of the shaded squares are proportional to study weight

Eleven studies [16, 34, 35, 37–39, 42, 43, 45, 48, 54] provided data on blood zeaxanthin levels in 851 patients with dementia compared with 2109 controls. A random-effect model was used because significant heterogeneity (*I*^2^ = 92.5%, *P* = 0.000) was observed across these studies. The results showed that blood zeaxanthin levels were substantially lower in patients with dementia compared with controls (SMD: -0.571; 95%CI: -0.910, -0.232) (Fig. 6). In the subgroup analysis, when stratified by disease type, a negative association was observed in both patients with AD, but not in patients with VaD and ID. When the data were stratified by sample source, a significant difference was observed in the plasma but not the serum subgroup (Supplemental Table 2). The meta-regression results indicated that these covariates had no significant impact on the heterogeneity among the studies (Supplemental Table 3). Sensitivity analysis indicated that the elimination of any study did not affect the combined SMD (Supplemental Fig. 1E). Egger’s test for the association between blood zeaxanthin levels and dementia showed no publication bias (*P* = 0.201) (Supplemental Table 4).

Two studies including 224 patients with MCI and 1684 controls, reported on plasma zeaxanthin levels [37, 51]. A single study [37] showed that plasma zeaxanthin levels in patients with MCI were notably lower than in controls (*P* < 0.01); however, one study [51] showed that patients with MCI had the same plasma zeaxanthin levels as controls. The pooled data suggested no significant between-group difference in plasma zeaxanthin levels (SMD: -0.317; 95%CI: -1.009, 0.376; *I*^*2*^ = 86.8%) (Supplemental Fig. 3).

### *β*-Cryptoxanthin

**Fig. 7:**
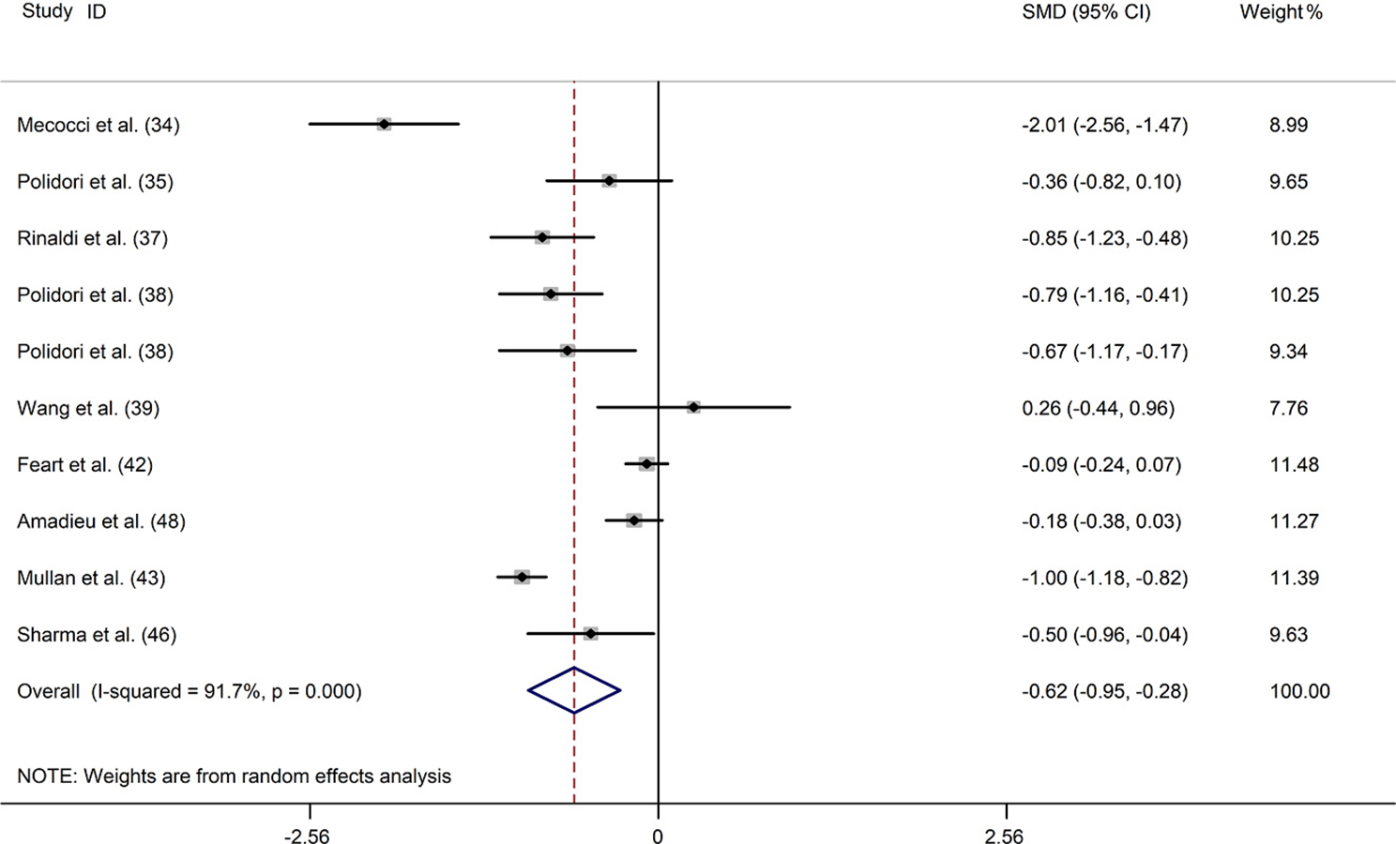
Forest plot of the relation between blood *β*-cryptoxanthin (µmol/L) and dementia and MCI, 2002–2020. The data are expressed as SMDs with 95%CIs. The overall effect is represented by a hollow diamond. The horizontal lines represent 95%CI. The sizes of the shaded squares are proportional to study weight

Nine papers [34, 35, 37–39, 42, 43, 46, 48] retrieved blood *β*-cryptoxanthin levels, including 780 patients with dementia and 2047 controls. The pooled findings showed that patients with dementia had lower blood *β*-cryptoxanthin levels than controls in a random-effect model (SMD: -0.617; 95%CI: -0.953, -0.281). Statistically, significant heterogeneity was noted among studies on blood *β-*cryptoxanthin levels (*I*^*2*^ = 91.7%, *P* = 0.000) (Fig. 7). The subgroup analyses showed that blood carotenoid levels were lower in patients with AD than in the controls, and the pooled SMD was significant both in the subgroups of serum and plasma (Supplemental Table 2). The meta-regression analyses showed that these covariates have no significant contribution to the between-study heterogeneity (Supplemental Table 3). In sensitivity analysis, leaving out any individual study did not cause large fluctuations in the combined SMD results (Supplemental Fig. 1F). Egger’s test for the association between blood *β*-cryptoxanthin levels and dementia detected no publication bias among these studies (*P* = 0. 492) (Supplemental Table 4).

Two publications reported plasma *β*-cryptoxanthin levels in 224 patients with MCI and 1684 controls [37, 51]. One study [37] measured significantly higher plasma levels of *β*-cryptoxanthin in patients with MCI than in controls (*P* < 0.001), while another study [51] reported appreciably lower plasma*β*-cryptoxanthin levels in patients with MCI than in controls (*P* < 0.05). The pooled data revealed that no significant differences were observed in plasma *β*-cryptoxanthin levels between patients with MCI and controls (SMD: -0.839; 95%CI: -2.553, 0.874; *I*^*2*^ = 97.9%) (Supplemental Fig. 3).

## Discussions

### Main findings

Given the inconsistency of the prior literature, the differences in blood carotenoid concentrations between patients with dementia and cognitively intact controls remain controversial. Meanwhile, information on carotenoid concentrations in the blood of patients with MCI is limited. Therefore, to address these critical issues, we performed the first extensive systematic review and meta-analysis to estimate their relationships and to provide a comprehensive critique of the evidence to establish whether relationships exist.

Emerging evidence suggests that oxidative damage is closely associated with the pathogenesis of dementia and MCI. Disadvantageous events in the brain, such as Aβ deposits, cerebral ischemia, or Lewy body formation, continuously damage mitochondrial function, produce reactive oxygen species (ROS), and induce oxidative stress [55]. ROS containing unpaired electrons with immense chemical reactivity actively reacts with intercellular proteins and lipids that are important for neuronal function, eventually propelling membrane lipid peroxidation and protein structural changes [56]. These alterations cause substantial neuronal dysfunction or loss, in turn forming neuropathological lesions that eventually contribute to dementia onset [57]. Consequently, a reduction in oxidative stress damage is a vital protective mechanism against dementia. Carotenoids with numerous conjugated olefinic bonds in their molecules can easily provide ROS with electrons and yield an inactive p-π conjugated system, thus quenching ROS and terminating harmful oxidative chain reactions [58]. Owning to the structural lipophilicity, several carotenoids allow them to traverse the blood-brain barrier into the brain, wherein they enable the embedding of neuronal membranes, further participating in scavenging ROS and avoiding the oxidation of lipid precursor membrane components [59]. Additionally, carotenoids are involved in the activation of antioxidant signaling pathways and upregulation of downstream antioxidant enzymes, thereby forming antioxidant defenses to combat dementia [60, 61]. Therefore, sustaining higher blood carotenoid levels appears to be key in protecting neurons against oxidative stress, which is also beneficial for preventing and improving dementia and MCI.

In our study, by incorporating the data of all included studies, we attained an answer for our research question: in comparison with cognitively normal controls, patients with dementia had a systematic deficiency in levels of blood carotenoids (*P* < 0.05). Several studies enabled us to provide consistent evidence of the correlation between lower blood carotenoid levels and dementia. A possible internal pathophysiological cause may be that patients with dementia have to consume more antioxidants, including carotenoids than normal cognitive controls to counteract excessive ROS production, which results in abnormal blood carotenoid changes [62]. From external cause, lower blood carotenoid levels in patients with dementia might result from poor dietary intake of fruits and vegetables. Unfortunately, it is very difficult to identify this issue as exists some degree of potential confounders. Several studies have suggested that patients with dementia have poor diets lacking in fruit and vegetables [63, 64]. However, in this study, most of the cited studies established a baseline prior to clinical studies to largely exclude the influence of carotenoid dietary intake on the comparison between patients with dementia and controls using dietary questionnaires or vegetables/fruits or carotenoid supplementation restriction. Meanwhile, we have to admit that these questionnaire investigations suffer from confounding bias on the long-term dietary habits and the accuracy of recent food/supplement compositions among participants. No matter whether the blood carotenoid deficits were caused by excessive anti-oxidative consumption or possible insufficient intakes and unbalanced diets, patients with dementia should be described as having special carotenoid-rich dietary requirements.

We further analyzed blood carotenoid levels in patients with MCI. We observed that individuals with MCI only possessed lower blood *α*-carotene, *β*-carotene, and lutein levels as compared to controls (*P* < 0.05). This evidence suggests that not all blood carotenoid levels reflect the condition of MCI. A major cause of these results might be the insufficient number of available publications. Another possibility is that the decrease in blood carotenoid levels is not typical in the early and mid-stages of dementia. Therefore, they could not identify sensitive biomarkers for assessing MCI. However, after pooling the overall carotenoid levels in patients with MCI, we found that the pooled SMD for MCI was − 0.650 (-1.045, -0.252), suggesting a tendency toward lower carotenoid levels (Supplemental Fig. 3). In this sense, our data indicate that earlier intervention with carotenoid supplementation in patients with MCI could result in better therapeutic effects. Consistent with this finding, a recent trial reported a significant increase in cognitive function measures following the use of avocado supplements for 6months in patients with MCI, which may be explained by the fact that avocado is rich in multiple carotenoids, and its consumption may increase blood carotenoid levels (ClinicalTrials.gov Identifier: NCT01620567). In 2020, another cognitive impairment study (CARES) demonstrated that a 12-month combined nutritional intervention ofω-3FAs, xanthophyll carotenoids, and vitamin E were able to restore cognitive performance and delay memory impairment in patients with MCI [65]. Notably, because of the relatively small number of studies, the results of the specified subgroup analysis should be interpreted cautiously, and more studies are needed for further assessment.

Furthermore, subgroup analyses were conducted to determine whether the results could be altered according to the different specified subgroups. Subgroup analysis of disease type showed that except for *β*-carotene, the concentrations of the other five carotenoids in the blood were significantly low in patients with AD and VaD (*P* < 0.05). Similarly, a previous meta-analytical study reported lower plasma levels of carotenes in individuals with AD as compared to controls [66]. Our observations not only corroborated their findings but also expanded their scope of applicability to the association between plasma/serum carotenoid levels and VaD. Moreover, we pooled their data and discovered that ID was possibly linked with lower blood carotenoid levels (SMD: -0.284; 95%CI: -0.464, -0.101) (Supplemental Fig. 4). Additionally, we assessed the impact of the sample source on the results. Generally, the concentrations of most compounds in plasma and serum samples should not differ according to pharmacokinetic theory. However, subgroup analysis of sample sources showed that lycopene, *α*-carotene, *β*-carotene, lutein, zeaxanthin, and *β*-cryptoxanthin levels were considerably lower in the plasma of patients with dementia. In contrast, no significant difference was observed in *β*-carotene and zeaxanthin levels in the serum samples. This result was attributed to the limited number of serum samples used for subgroup analyses in the included studies (only six studies), thereby leading to imprecision and inconsistency of the subgroup analyses.

Our meta-analysis has several limitations, which should be acknowledged when interpreting findings. Although this study found evidence of lower blood carotenoid levels in patients with dementia as compared to controls, the relationship between blood carotenoid levels and MCI is not entirely clear. Hence, more prospective studies should be conducted to provide greater evidence of the role of blood carotenoid levels at the MCI stage. Additionally, there are limited studies supplying data regarding blood carotenoid levels in patients with VaD and other dementias. Moreover, because of the lack of special forms of diet, body mass index, and Mini-Mental State Exam scores, we could not directly infer whether nutrient status and dementia severity affect blood carotenoid levels. Therefore, further in-depth studies are needed to identify potential associations. It would also be worth elucidating the cause of the systematic deficiency in blood carotenoids in individuals with dementia.

## Conclusion

In conclusion, this meta-analysis found that patients with dementia had significantly lower blood carotenoid levels, despite high heterogeneity. However, we could not draw clear and stable evidence on blood carotenoid levels in patients with MCI, because of less severe disease progression and insufficient data. Therefore, further well-designed and large-scale studies are necessary to reduce the heterogeneity and consolidate our conclusions. As a final recommendation, given their convenience and safety, the intake of carotenoid-rich food or medicine supplements may be beneficial to reducing the risk of dementia in the future.

## Electronic supplementary material

Below is the link to the electronic supplementary material.

Supplemental Table 1. Quality assessment according to Newcastle-Ottawa Scale (NOS).
Supplemental Table 2. Subgroup analysis for SMD of blood carotenoid levels in patients with dementia.
Supplemental Table 3. Meta-regression for SMD of blood carotenoid levels in patients with dementia.
Supplemental Table 4. Egger’s test of blood carotenoid levels in patients with dementia.
Supplemental Figure 1. Forest plot of sensitivity analysis in the studies.
Supplemental Figure 2. Funnel plot of detailing publication bias in the studies.
Supplemental Figure 3. Forest plot of blood carotenoid levels between patients with MCI and HC subjects.
Supplemental Figure 4. Forest plot of blood carotenoid levels between patients with indefinite dementia (ID) and HC subjects.

## Data Availability

All data and materials generated or analyzed in this study are included in this published article and appendix file.

## Abbreviations

ABCB1: ATP binding cassette B1
AD: Alzheimer’s disease
CI: Confidence intervals
ID: Indefinite dementia
HDL: LBD
High-density lipoprotein: Lewy-body dementia
MCI: Mild cognitive impairment
NOS: Newcastle-Ottawa Scale
ROS: Reactive oxygen species
SD: Standard deviations
SMD: Standardized mean differences
VaD: Vascular dementia

## References

[1] Grand JH, Caspar S, Macdonald SW (2011). Clinical features and multidisciplinary approaches to dementia care. J Multidiscip Healthc.

[2] Choromańska M, Klimiuk A, Kostecka-Sochoń P, Wilczyńska K, Kwiatkowski M, Okuniewska N, Waszkiewicz N, Zalewska A, Maciejczyk M (2017). Antioxidant defence, oxidative stress and oxidative damage in saliva, plasma and erythrocytes of dementia patients. Can salivary AGE be a marker of dementia?. Int J Mol Sci.

[3] Jack CR, Bennett DA, Blennow K, Carrillo MC, Dunn B, Haeberlein SB, Holtzman DM, Jagust W, Jessen F, Karlawish J (2018). NIA-AA Research Framework: toward a biological definition of Alzheimer’s disease. Alzheimers Dement.

[4] Feldman HH, Ferris S, Winblad B, Sfikas N, Mancione L, He Y, Tekin S, Burns A, Cummings J, del Ser T (2007). Effect of rivastigmine on delay to diagnosis of Alzheimer’s disease from mild cognitive impairment: the InDDEx study. Lancet Neurol.

[5] Islam BU, Jabir NR, Tabrez S (2019). The role of mitochondrial defects and oxidative stress in Alzheimer’s disease. J Drug Target.

[6] Hammond BR, Renzi LM, Carotenoids (2013). Adv Nutr.

[7] Shete V, Quadro L (2013). Mammalian metabolism of beta-carotene: gaps in knowledge. Nutrients.

[8] Nagao A (2014). Bioavailability of dietary carotenoids: intestinal absorption and metabolism. Jarq-J Agr Res Q.

[9] Britton G (1995). Structure and properties of carotenoids in relation to function. Faseb J.

[10] Craft NE, Haitema TB, Garnett KM, Fitch KA, Dorey CK (2004). Carotenoid, tocopherol, and retinol concentrations in elderly human brain. J Nutr Health Aging.

[11] Nishino A, Yasui H, Maoka T (2016). Reaction of paprika carotenoids, capsanthin and capsorubin, with reactive oxygen species. J Agric Food Chem.

[12] Lim KG, Varatharajan R, Muthuraman A. The attenuating effect of Beta-carotene on Streptozotocin Induced Diabetic Vascular dementia symptoms in rats. Molecules. 2022;27(13). 10.3390/molecules27134293. 10.3390/molecules27134293 PMC9268603 35807538

[13] Dhas N, Mehta T (2020). Cationic biopolymer functionalized nanoparticles encapsulating lutein to attenuate oxidative stress in effective treatment of Alzheimer’s disease: a non-invasive approach. Int J Pharm.

[14] Sachdeva AK, Chopra K (2015). Lycopene abrogates Aβ(1–42)-mediated neuroinflammatory cascade in an experimental model of Alzheimer’s disease. J Nutr Biochem.

[15] Yin Q, Ma Y, Hong Y, Hou X, Chen J, Shen C, Sun M, Shang Y, Dong S, Zeng Z (2014). Lycopene attenuates insulin signaling deficits, oxidative stress, neuroinflammation, and cognitive impairment in fructose-drinking insulin resistant rats. Neuropharmacology.

[16] Nolan JM, Loskutova E, Howard AN, Moran R, Mulcahy R, Stack J, Bolger M, Dennison J, Akuffo KO, Owens N (2014). Macular pigment, visual function, and macular disease among subjects with Alzheimer’s disease: an exploratory study. J Alzheimers Dis.

[17] Li X, Zhang P, Li H, Yu H, Xi Y (2022). The protective effects of zeaxanthin on Amyloid-β peptide 1-42-induced impairment of learning and memory ability in rats. Front Behav Neurosci.

[18] Zhong Q, Sun W, Qin Y, Xu H. Association of dietary α-carotene and β-carotene intake with low cognitive performance in older adults: a cross-sectional study from the national health and nutrition examination survey. Nutrients. 2023;15(1). 10.3390/nu15010239. 10.3390/nu15010239 PMC9823947 36615894

[19] Davinelli S, Ali S, Solfrizzi V, Scapagnini G, Corbi G. Carotenoids and cognitive outcomes: a meta-analysis of randomized intervention trials. Antioxid (Basel Switzerland). 2021;10(2). 10.3390/antiox10020223. 10.3390/antiox10020223 PMC7913239 33540909

[20] Yuan C, Chen H, Wang Y, Schneider JA, Willett WC, Morris MC (2021). Dietary carotenoids related to risk of incident Alzheimer dementia (AD) and brain AD neuropathology: a community-based cohort of older adults. Am J Clin Nutr.

[21] Debelo H, Novotny JA, Ferruzzi MG, Vitamin A (2017). Adv Nutr.

[22] Wang W, Shinto L, Connor WE, Quinn JF (2008). Nutritional biomarkers in Alzheimer’s disease: the association between carotenoids, n-3 fatty acids, and dementia severity. J Alzheimers Dis.

[23] Engelhart MJ, Ruitenberg A, Meijer J, Kiliaan A, van Swieten JC, Hofman A, Witteman JC, Breteler MM (2005). Plasma levels of antioxidants are not associated with Alzheimer’s disease or cognitive decline. Dement Geriatr Cogn Disord.

[24] Qu M, Shi H, Wang K, Wang X, Yu N, Guo B (2021). The associations of plasma/serum carotenoids with alzheimer’s disease: a systematic review and meta-analysis. J Alzheimers Dis.

[25] Koch M, Furtado JD, Cronjé HT, DeKosky ST, Fitzpatrick AL, Lopez OL, Kuller LH, Mukamal KJ, Jensen MK (2021). Plasma antioxidants and risk of dementia in older adults. Alzheimers Dement (N Y).

[26] Liberati A, Altman DG, Tetzlaff J, Mulrow C, Gøtzsche PC, Ioannidis JP, Clarke M, Devereaux PJ, Kleijnen J, Moher D (2009). The PRISMA statement for reporting systematic reviews and meta-analyses of studies that evaluate health care interventions: explanation and elaboration. PLoS Med.

[27] Hozo SP, Djulbegovic B, Hozo I (2005). Estimating the mean and variance from the median, range, and the size of a sample. BMC Med Res Methodol.

[28] Wells G, Shea B, O’Connell D, Peterson j, Welch V, Losos M, Tugwell P. The Newcastle-Ottawa Scale (NOS) for assessing the quality of non-randomized studies in meta-analysis. 2000.

[29] Higgins JPT, Thompson SG (2002). Quantifying heterogeneity in a meta-analysis. Stat Med.

[30] Higgins JP, Thompson SG (2004). Controlling the risk of spurious findings from meta-regression. Stat Med.

[31] Tobias A (1999). Assessing the influence of a single study in the meta-anyalysis estimate. Stata Tech Bull.

[32] Egger M, Davey Smith G, Schneider M, Minder C (1997). Bias in meta-analysis detected by a simple, graphical test. BMJ (Clinical research ed).

[33] Foy CJ, Passmore AP, Vahidassr MD, Young IS, Lawson JT (1999). Plasma chain-breaking antioxidants in Alzheimer’s disease, vascular dementia and Parkinson’s disease. QJM.

[34] Mecocci P, Polidori MC, Cherubini A, Ingegni T, Mattioli P, Catani M, Rinaldi P, Cecchetti R, Stahl W, Senin U (2002). Lymphocyte oxidative DNA damage and plasma antioxidants in Alzheimer disease. Arch Neurol.

[35] Polidori MC, Mecocci P (2002). Plasma susceptibility to free radical-induced antioxidant consumption and lipid peroxidation is increased in very old subjects with Alzheimer disease. J Alzheimers Dis.

[36] Quinn J, Suh J, Moore MM, Kaye J, Frei B (2003). Antioxidants in Alzheimer’s disease-vitamin C delivery to a demanding brain. J Alzheimers Dis.

[37] Rinaldi P, Polidori MC, Metastasio A, Mariani E, Mattioli R, Cherubini A, Catani M, Cecchetti R, Senin U, Mecocci P (2003). Plasma antioxidants are similarly depleted in mild cognitive impairment and in Alzheimer’s disease. Neurobiol Aging.

[38] Polidori MC, Mattioli P, Aldred S, Cecchetti R, Stahl W, Griffiths H, Senin U, Sies H, Mecocci P (2004). Plasma antioxidant status, immunoglobulin g oxidation and lipid peroxidation in demented patients: relevance to Alzheimer disease and vascular dementia. Dement Geriatr Cogn Disord.

[39] Wang W, Shinto L, Connor WE, Quinn JF (2008). Nutritional biomarkers in Alzheimer’s disease: the association between carotenoids, n-3 fatty acids, and dementia severity. J Alzheimers Dis.

[40] von Arnim CAF, Herbolsheimer F, Nikolaus T, Peter R, Biesalski HK, Ludolph AC, Riepe M, Nagel G, Acti FEUSG (2012). Dietary antioxidants and dementia in a population-based case-control study among older people in South Germany. J Alzheimers Dis.

[41] Giavarotti L, Simon KA, Azzalis LA, Fonseca FLA, Lima AF, Freitas MCV, Brunialti MKC, Salomao R, Moscardi AAVS, Montano MBMM, et al. Mild systemic oxidative stress in the subclinical stage of Alzheimer’s disease. Oxid Med Cell Longev. 2013;609019. 10.1155/2013/609019. 10.1155/2013/609019 PMC3880752 24454987

[42] Feart C, Letenneur L, Helmer C, Samieri C, Schalch W, Etheve S, Delcourt C, Dartigues J-F, Barberger-Gateau P (2016). Plasma carotenoids are inversely associated with dementia risk in an elderly french cohort. J Gerontol A Biol Sci Med Sci.

[43] Mullan K, Williams MA, Cardwell CR, McGuinness B, Passmore P, Silvestri G, Woodside JV, McKay GJ (2017). Serum concentrations of vitamin E and carotenoids are altered in Alzheimer’s disease: a case-control study. Alzheimers Dement.

[44] Boccardi V, Arosio B, Cari L, Bastiani P, Scamosci M, Casati M, Ferri E, Bertagnoli L, Ciccone S, Rossi PD (2019). Beta-carotene, telomerase activity and Alzheimer’s disease in old age subjects. Eur J Nutr.

[45] Dias IHK, Polidori MC, Li L, Weber D, Stahl W, Nelles G, Grune T, Griffiths HR (2014). Plasma levels of HDL and carotenoids are lower in dementia patients with vascular comorbidities. J Alzheimers Dis.

[46] Sharma A, Weber D, Raupbach J, Dakal TC, Fließbach K, Ramirez A, Grune T, Wüllner U (2020). Advanced glycation end products and protein carbonyl levels in plasma reveal sex-specific differences in Parkinson’s and Alzheimer’s disease. Redox Biol.

[47] Jimenez-Jimenez FJ, Molina JA, de Bustos F, Orti-Pareja M, Benito-Leon J, Tallon-Barranco A, Gasalla T, Porta J, Arenas J (1999). Serum levels of beta-carotene, alpha-carotene and vitamin A in patients with Alzheimer’s disease. Eur J Neurol.

[48] Amadieu C, Lefevre-Arbogast S, Delcourt C, Dartigues JF, Helmer C, Feart C, Samieri C (2017). Nutrient biomarker patterns and long-term risk of dementia in older adults. Alzheimers Dement.

[49] Ayromlou H, Pourvahed P, Jahanjoo F, Dolatkhah H, Shakouri SK, Dolatkhah N (2018). Dietary and serum level of antioxidants in the elderly with mild impaired and normal cognitive function: a case-control study. Iran Red Crescent Me.

[50] Schippling S, Kontush A, Arlt S, Buhmann C, Sturenburg HJ, Mann U, Muller-Thomsen T, Beisiegel U (2000). Increased lipoprotein oxidation in Alzheimer’s disease. Free Radical Biol Med.

[51] Rietman ML, Spijkerman AMW, Wong A, van Steeg H, Burkle A, Moreno-Villanueva M, Sindlinger T, Franceschi C, Grubeck-Loebenstein B, Bernhardt J (2019). Antioxidants linked with physical, cognitive and psychological frailty: analysis of candidate biomarkers and markers derived from the MARK-AGE study. Mech Ageing Dev.

[52] Sinclair AJ, Bayer AJ, Johnston J, Warner C, Maxwell SR (1998). Altered plasma antioxidant status in subjects with Alzheimer’s disease and vascular dementia. Int J Geriatr Psych.

[53] Mangialasche F, Xu W, Kivipelto M, Costanzi E, Ercolani S, Pigliautile M, Cecchetti R, Baglioni M, Simmons A, Soininen H (2012). Tocopherols and tocotrienols plasma levels are associated with cognitive impairment. Neurobiol Aging.

[54] Nolan JM, Loskutova E, Howard A, Mulcahy R, Moran R, Stack J, Bolger M, Coen RF, Dennison J, Akuffo KO (2015). The impact of supplemental macular carotenoids in Alzheimer’s disease: a randomized clinical trial. J Alzheimers Dis.

[55] Bennett S, Grant MM, Aldred S (2009). Oxidative stress in vascular dementia and Alzheimer’s disease: a common pathology. J Alzheimers Dis.

[56] Cervellati C, Romani A, Seripa D, Cremonini E, Bosi C, Magon S, Passaro A, Bergamini CM, Pilotto A, Zuliani G (2014). Oxidative balance, homocysteine, and uric acid levels in older patients with late onset Alzheimer’s disease or vascular dementia. J Neurol Sci.

[57] Kosicek M, Hecimovic S (2013). Phospholipids and Alzheimer’s disease: alterations, mechanisms and potential biomarkers. Int J Mol Sci.

[58] Liaaen-Jensen S, Lutnaes BF. Structure and properties of carotenoid cations, 2008;4.

[59] Widomska J, Welc R, Gruszecki WI (2019). The effect of carotenoids on the concentration of singlet oxygen in lipid membranes. Biochim Biophys Acta Biomembr.

[60] Wang J, Li L, Wang Z, Cui Y, Tan X, Yuan T, Liu Q, Liu Z, Liu X (2018). Supplementation of lycopene attenuates lipopolysaccharide-induced amyloidogenesis and cognitive impairments via mediating neuroinflammation and oxidative stress. J Nutr Biochem.

[61] Liu T, Liu WH, Zhao JS, Meng FZ, Wang H (2017). Lutein protects against β-amyloid peptide-induced oxidative stress in cerebrovascular endothelial cells through modulation of Nrf-2 and NF-κb. Cell Biol Toxicol.

[62] Bourdel-Marchasson I, Delmas-Beauvieux MC, Peuchant E, Richard-Harston S, Decamps A, Reignier B, Emeriau JP, Rainfray M (2001). Antioxidant defences and oxidative stress markers in erythrocytes and plasma from normally nourished elderly Alzheimer patients. Age Ageing.

[63] Morley JE (2010). Nutrition and the brain. Clin Geriatr Med.

[64] Mi W, van Wijk N, Cansev M, Sijben JW, Kamphuis PJ (2013). Nutritional approaches in the risk reduction and management of Alzheimer’s disease. Nutrition.

[65] Power R, Nolan JM, Prado-Cabrero A, Coen R, Roche W, Power T, Howard AN, Mulcahy R. Targeted nutritional intervention for patients with mild cognitive impairment: the cognitive impAiRmEnt study (CARES) trial 1. J Pers Med. 2020;10(2). 10.3390/jpm10020043. 10.3390/jpm10020043 PMC7354621 32466168

[66] Mullan K, Cardwell CR, McGuinness B, Woodside JV, McKay GJ (2018). Plasma antioxidant status in patients with Alzheimer’s Disease and cognitively intact elderly: a meta-analysis of case-control studies. J Alzheimers Dis.

