# Supplementary material for: Low blood carotenoid status in dementia and mild cognitive impairment: A systematic review and meta-analysis
Source: BMC Geriatr. 2023 Mar 30;23:195. doi: 10.1186/s12877-023-03900-7 (PMC10064563; doi:10.1186/s12877-023-03900-7)
Supplement: Supplementary file 1 — Supplemental Table 1. Quality assessment according to Newcastle-Ottawa Scale (NOS). Supplemental Table 2. Subgroup analysis for SMD of blood carotenoid levels in patients with dementia. Supplemental Table 3. Meta-regression for SMD of blood carotenoid levels in patients with dementia. Supplemental Table 4. Egger’s test of blood carotenoid levels in patients with dementia. Supplemental Figure 1. Forest plot of sensitivity analysis in the studies. Supplemental Figure 2. Funnel plot of detailing publication bias in the studies. Supplemental Figure 3. Forest plot of blood carotenoid levels between patients with MCI and HC subjects. Supplemental Figure 4. Forest plot of blood carotenoid levels between patients with indefinite dementia (ID) and HC subjects. [file 12877_2023_3900_MOESM1_ESM.docx]

**Low blood carotenoid status in dementia and mild cognitive impairment: a systematic review and meta-analysis**

**Lin Wang et al. Online Supplementary Materials**

**Supplemental Table 1 Quality assessment according to Newcastle-Ottawa Scale (NOS).**

| **Author(reference)** | **Country** | **Selection** | | | | **Comparability** | | **Exposure** | | | **Overall Quality** |
| --- | --- | --- | --- | --- | --- | --- | --- | --- | --- | --- | --- |
|  |  | 1 | 2 | 3 | 4 | 5A | 5B | 6 | 7 | 8 |  |
| Sinclair et al. (52) | UK | * |  |  | * | * | * | * | * |  | 6 |
| Foy et al.(33) | UK | * | * |  | * | * | * | * | * | * | 8 |
| Jimenez et al. (47) | Spain | * |  |  | * | * | * | * | * | * | 7 |
| Schippling et al.(50) | Germany | * | * |  | * | * | * | * | * | * | 8 |
| Mecocci et al. (34) | Italy | * |  |  | * | * | * | * | * | * | 7 |
| Polidori et al. (35) | France | * |  |  | * | * | * | * | * | * | 7 |
| Quinn et al. (36) | USA | * | * |  | * | * | * | * | * |  | 7 |
| Rinaldi et al. (37) | Italy | * | * | * | * | * | * | * | * |  | 8 |
| Polidori et al. (38) | Germany | * | * |  | * | * | * | * | * |  | 7 |
| Wang et al. (39) | USA | * | * |  | * | * | * | * | * | * | 8 |
| von Arnim et al. (40) | Germany | * | * | * | * | * | * | * | * | * | 9 |
| Giavarotti et al. (41) | Brazil | * |  |  | * | * | * | * | * | * | 7 |
| Nolan et al. (54) | Italy | * | * | * | * | * | * | * | * | * | 9 |
| Nolan et al. (55) | Italy | * | * |  | * | * | * | * | * |  | 7 |
| Feart et al. (42) | France | * | * |  | * | * | * | * | * | * | 8 |
| Amadieu et al. (48) | France | * | * | * | * | * | * | * | * | * | 9 |
| Mullan et al. (43) | UK | * | * |  | * | * | * | * | * | * | 8 |
| Ayromlou et al. (49) | Iran | * | * |  | * | * | * | * | * |  | 7 |
| Boccardi et al. (44) | Italy | * | * |  | * | * | * | * | * | * | 8 |
| Rietman et al. (51) | Netherlands | * |  |  | * | * | * | * | * | * | 7 |
| Diasa et al. (45) | Germany | * | * | * | * | * | * | * | * | * | 9 |
| Mangialasche (53) | European | * | * | * | * | * | * | * | * | * | 9 |
| Sharma (46) | Germany | * | * | * | * | * | * | * | * | * | 9 |

A study can be awarded a maximum of one star for each numbered item within the Selection and Exposure categories and a maximum of two stars for Comparability. Scores for low (0-3), moderate (4-6), and high-quality studies (7-9) were assigned.

**Supplemental Table** **2 Subgroup analysis for SMD of blood carotenoid levels in patients with dementia**

| **Carotenoids** | **Variable** | **No. of  Studies** | **SMD (95% CI)** | **Test of SMD=0** | | **Heterogeneity** | |
| --- | --- | --- | --- | --- | --- | --- | --- |
|  |  |  |  | **Z** | ***P* for Z** | ***I*^2^ (%)** | ***P* for *I*^2^** |
| Lycopene | All studies | 16 | -0.521, (-0.741, -0.301) | 4.63 | 0.000 | 86.1 | 0.000 |
|  | Dementia type |  |  |  |  |  |  |
|  | AD | 12 | -0.622, (-0.961, -0.283) | 3.60 | 0.000 | 87.5 | 0.000 |
|  | ID | 3 | -0.401, (-0.811, 0.009) | 1.92 | 0.055 | 91.3 | 0.000 |
|  | VaD | 3 | -0.265, (-0.525, -0.006) | 2.00 | 0.041 | 0.0 | 0.486 |
|  | Sample source |  |  |  |  |  |  |
|  | Serum | 3 | -0.630, (-0.965, -0.295) | 3.68 | 0.000 | 75.4 | 0.017 |
|  | Plasma | 15 | -0.498, (-0.770, -0.226) | 3.59 | 0.000 | 87.0 | 0.000 |
|  | | | | | | | |
| α-Carotene | All studies | 13 | -0.489, (-0.697, -0.281) | 4.60 | 0.000 | 81.6 | 0.000 |
|  | Dementia type |  |  |  |  |  |  |
|  | AD | 11 | -0.536, (-0.769, -0.302) | 4.50 | 0.000 | 71.5 | 0.000 |
|  | ID | 2 | -0.051, (-0.182, 0.079) | 0.77 | 0.439 | 9.8 | 0.292 |
|  | VaD | 2 | -0.810, (-1.215, -0.406) | 3.92 | 0.000 | 33.3 | 0.219 |
|  | Sample source |  |  |  |  |  |  |
|  | Plasma | 13 | -0.528, (-0.797, -0.259) | 4.05 | 0.000 | 83.1 | 0.000 |
|  | Serum | 2 | -0.398, (-0.699, -0.096) | 2.59 | 0.010 | 48.0 | 0.165 |
|  |  |  |  |  |  |  |  |
| β-Carotene | All studies | 17 | -0.476, (-0.784, -0.168) | 3.03 | 0.002 | 93.9 | 0.000 |
|  | Dementia type |  |  |  |  |  |  |
|  | AD | 14 | -0.413, (-0.763, -0.062) | 2.31 | 0.021 | 91.4 | 0.000 |
|  | ID | 3 | -0.821, (-1.788, 0.145) | 1.67 | 0.096 | 98.3 | 0.000 |
|  | VaD | 3 | -0.390, (-1.724, 0.943) | 0.57 | 0.566 | 95.1 | 0.000 |
|  | Sample source |  |  |  |  |  |  |
|  | Plasma | 17 | -0.372, (-0.695, -0.049) | 2.26 | 0.024 | 92.5 | 0.000 |
|  | Serum | 3 | -1.035, (-2.159, 0.090) | 1.80 | 0.074 | 97.5 | 0.000 |
|  |  |  |  |  |  |  |  |
| Lutein | All studies | 11 | -0.516, (-0.753, -0.279) | 4.27 | 0.000 | 84.2 | 0.000 |
|  | Dementia type |  |  |  |  |  |  |
|  | AD | 8 | -0.619, (-0.945, -0.293) | 3.72 | 0.000 | 81.4 | 0.000 |
|  | ID | 2 | -0.111, (-0.234, 0.012) | 1.77 | 0.076 | 0.0 | 0.593 |
|  | VaD | 2 | -1.100, (-1.618, -0.583) | 4.17 | 0.000 | 84.1 | 0.000 |
|  | Sample source |  |  |  |  |  |  |
|  | Plasma | 9 | -0.564, (-0.898, -0.229) | 3.30 | 0.001 | 88.4 | 0.000 |
|  | Serum | 3 | -0.354, (-0.505, -0.203) | 4.59 | 0.000 | 0.0 | 0.723 |
|  |  |  |  |  |  |  |  |
| Zeaxanthin | All studies | 11 | -0.571, (-0.910, -0.232) | 3.30 | 0.001 | 92.5 | 0.000 |
|  | Dementia type |  |  |  |  |  |  |
|  | AD | 8 | -0.704, (-1.270, -0.138) | 2.44 | 0.015 | 93.8 | 0.000 |
|  | ID | 2 | 0.000, (-0.123, 0.123) | 0.00 | 1.000 | 0.0 | 1.000 |
|  | VaD | 2 | -0.676, (-1.492, 0.141) | 1.62 | 0.105 | 82.3 | 0.018 |
|  | Sample source |  |  |  |  |  |  |
|  | Plasma | 9 | -0.652 (-1.120, -0.184) | 2.73 | 0.006 | 94.1 | 0.000 |
|  | Serum | 3 | -0.304 (-0.726, 0.118) | 1.41 | 0.158 | 73.1 | 0.024 |
| *β*-Cryptoxanthin | All studies | 9 | -0.617, (-0.953, -0.281) | 3.60 | 0.000 | 91.7 | 0.000 |
|  | Dementia type |  |  |  |  |  |  |
|  | AD | 7 | -0.704, (-1.270, -0.138) | 4.18 | 0.000 | 83.5 | 0.000 |
|  | ID | 2 | 0.000, (-0.123, 0.123) | 1.89 | 0.059 | 0.0 | 0.480 |
|  | VaD | 1 | -0.676, (-1.492, 0.141) | 2.62 | 0.009 | - | - |
|  | Sample source |  |  |  |  |  |  |
|  | Plasma | 9 | -0.566 (-0.896, -0.236) | 3.36 | 0.001 | 88.0 | 0.000 |
|  | Serum | 1 | -1.000 (-1.177, -0.823) | 11.09 | 0.000 | - | - |

Abbreviation: AD, Alzheimer's disease; CI, 95% confidence interval; ID, indefinite dementia; SMD, standardized mean difference.

**Supplemental Table** **3 Meta-regression for SMD of blood carotenoid levels in patients with dementia**

| **Carotenoids** | **Covariates** | **Coefficients** | **95% CI** | **Std. Err.** | ***P*** | **Adj *I*^2^ (%)** |
| --- | --- | --- | --- | --- | --- | --- |
| Lycopene | Mean age (years)-Patients | -0.074 | -0.155, 0.006 | 0.037 | 0.067 | 18.32 |
|  | Male (%)-Patients | 0.017 | -0.003, 0.038 | 0.010 | 0.094 | 13.24 |
|  | Country-Patients | 0.000 | -0.169, 0.169 | 0.080 | 0.997 | -8.32 |
|  | NOS score | 0.330 | -0.071, 0.730 | 0.189 | 0.100 | 14.96 |
|  | Diagnostic criteria-Patients | 0.030 | -0.242, 0.303 | 0.128 | 0.816 | -7.54 |
|  |  |  |  |  |  |  |
| α-Carotene | Mean age (years)-Patients | -0.022 | -0.072, 0.028 | 0.023 | 0.354 | -1.21 |
|  | Male (%)-Patients | 0.004 | -0.009, 0.018 | 0.006 | 0.471 | -5.43 |
|  | Country-Patients | -0.069 | -0.213, 0.075 | 0.067 | 0.317 | -0.19 |
|  | NOS score | 0.100 | -0.287, 0.486 | 0.178 | 0.587 | -5.18 |
|  | Diagnostic criteria-Patients | 0.023 | -0.212, 0.258 | 0.109 | 0.837 | -10.22 |
|  |  |  |  |  |  |  |
| *β*-Carotene | Mean age (years)-Patients | -0.020 | -0.126, 0.086 | 0.050 | 0.695 | -6.03 |
|  | Male (%)-Patients | -0.019 | -0.045, 0.007 | 0.012 | 0.134 | 11.12 |
|  | Country-Patients | -0.020 | -0.229, 0.189 | 0.099 | 0.841 | -6.04 |
|  | NOS score | -0.063 | -0.525, 0.400 | 0.220 | 0.779 | -5.15 |
|  | Diagnostic criteria-Patients | -0.032 | -0.328, 0.264 | 0.141 | 0.825 | -5.62 |
|  |  |  |  |  |  |  |
| Lutein | Mean age (years)-Patients | -0.050 | -0.166, 0.067 | 0.051 | 0.358 | -2.50 |
|  | Male (%)-Patients | 0.024 | 0.002, 0.047 | 0.010 | 0.036 | 40.63 |
|  | Country-Patients | 0.093 | -0.124, 0.310 | 0.097 | 0.365 | -1.70 |
|  | NOS score | 0.271 | -0.083, 0.626 | 0.159 | 0.119 | 20.84 |
|  | Diagnostic criteria-Patients | 0.041 | -0.272, 0.354 | 0.140 | 0.776 | -9.96 |
|  |  |  |  |  |  |  |
| Zeaxanthin | Mean age (years)-Patients | 0.078 | -0.089, 0.244 | 0.073 | 0.318 | 2.45 |
|  | Male (%)-Patients | -0.003 | -0.043, 0.038 | 0.018 | 0.884 | -11.92 |
|  | Country-Patients | 0.211 | -0.084, 0.508 | 0.132 | 0.143 | 13.30 |
|  | NOS score | 0.363 | -0.168, 0.894 | 0.238 | 0.158 | 12.67 |
|  | Diagnostic criteria-Patients | -0.026 | -0.482, 0.413 | 0.205 | 0.903 | -11.05 |
| *β*-Cryptoxanthin | Mean age (years)-Patients | 0.006 | -0.142, 0.154 | 0.063 | 0.922 | -18.94 |
|  | Male (%)-Patients | -0.010 | -0.045, 0.024 | 0.015 | 0.500 | -7.24 |
|  | Country-Patients | 0.091 | -0.227, 0.410 | 0.138 | 0.525 | -10.83 |
|  | NOS score | 0.328 | -0.229, 0.886 | 0.242 | 0.212 | 10.90 |
|  | Diagnostic criteria-Patients | 0.045 | -0.404, 0.493 | 0.194 | 0.823 | -13.82 |

Abbreviation: CI, 95% confidence interval; Std. Err.: standard error; SMD, standardized mean difference;

**Supplemental Table 4 Egger's test of blood carotenoid levels in patients with dementia**

| **Carotenoids** | **Std_Eff** | **Coef.** | **Std. Err.** | **t** | **P>\|t\|** | **[95%** **CI]** |
| --- | --- | --- | --- | --- | --- | --- |
| Lycopene | slope \| | -0.0873757 | 0.2124206 | -0.41 | 0.686 | -0.5376872, 0.3629358 |
|  | bias \| | -2.075819 | 1.38371 | -1.58 | 0.133 | -4.85664, 0.7050015 |
|  |  |  |  |  |  |  |
| *α*-Carotene | slope \| | -0.0099102 | 0.1781717 | -0.06 | 0.956 | -0.3948267, 0.3750063 |
|  | bias \| | -2.351474 | 1.136662 | -2.07 | 0.059 | -4.807084, 0.1041355 |
|  |  |  |  |  |  |  |
| *β*-Carotene | slope \| | -0.3662468 | 0.3317578 | -1.10 | 0.284 | -1.063244, 0.3307504 |
|  | bias \| | -0.6026771 | 2.035235 | -0.30 | 0.771 | -4.878548, 3.673193 |
|  |  |  |  |  |  |  |
| Lutein | slope \| | 0.0105692 | 0.198761 | 0.05 | 0.959 | -0.432298, 0.4534363 |
|  | bias \| | -2.710527 | 1.345038 | -2.02 | 0.072 | -5.707457, 0.2864034 |
|  |  |  |  |  |  |  |
| Zeaxanthin | slope \| | 0.3565004 | 0.2605586 | 1.37 | 0.201 | -0.2240604, 0.9370612 |
|  | bias \| | -4.616289 | 1.753518 | -2.63 | 0.025 | -8.523372, -0.7092069 |
|  |  |  |  |  |  |  |
| *β*-Cryptoxanthin | slope \| | -0.2412853 | 0.3353355 | -0.72 | 0.492 | -1.01457, 0.5319998 |
|  | bias \| | -2.010076 | 2.3816 | -0.84 | 0.423 | -7.502056, 3.481905 |

Abbreviation: CI, 95% confidence interval; Coef, coefficient; Std Err.: standard error; SMD, standardized mean difference;

**Supplemental Figures**

**
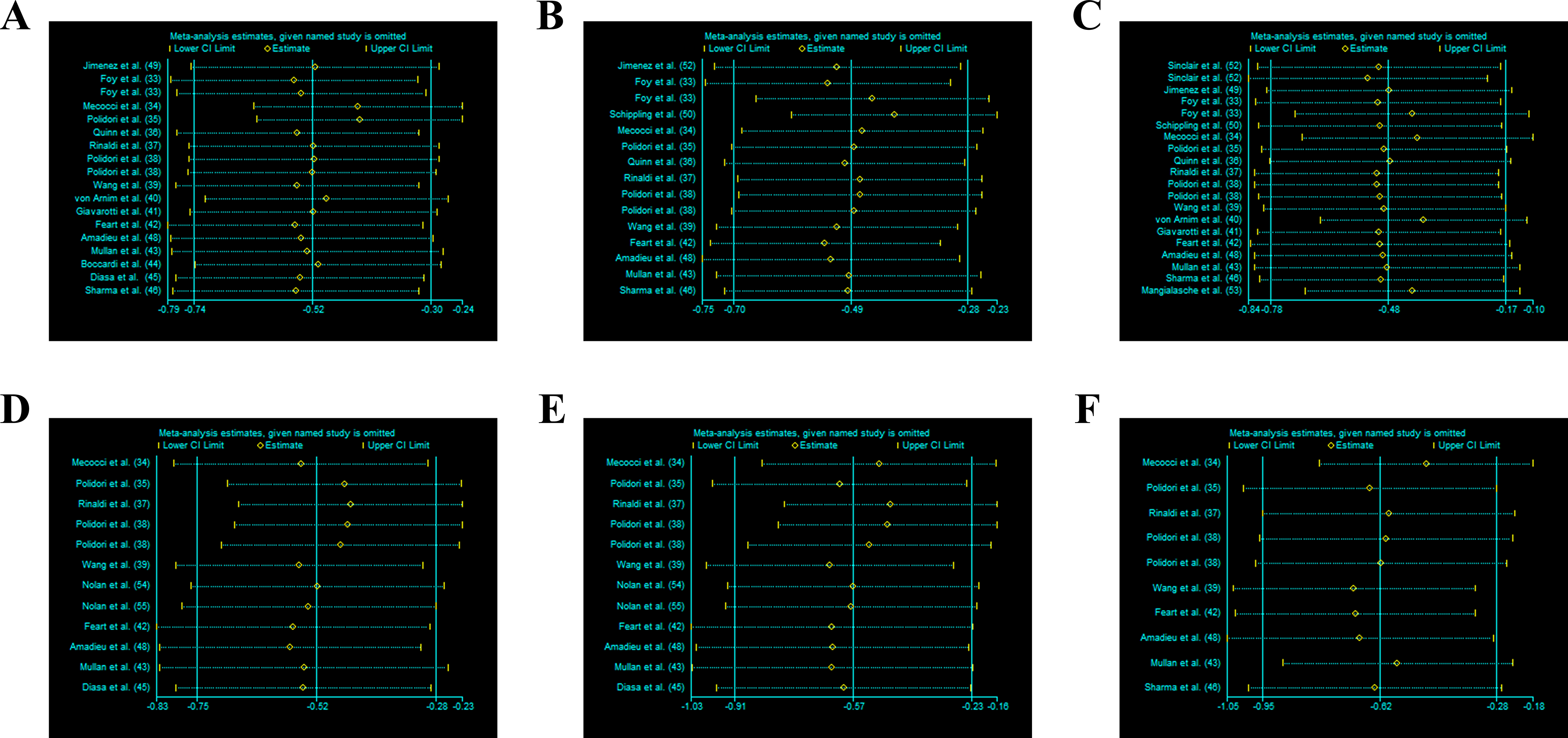
**

**Supplemental Figure 1 Forest plot of sensitivity analysis in the studies.** (A) blood lycopene levels in patients with dementia; (B) blood α-carotene levels in patients with dementia; (C) blood β-carotene levels in patients with dementia; (D) blood lutein levels in patients with dementia; (E) blood zeaxanthin levels in patients with dementia; (F) blood zeaxanthin levels in patients with dementia. The influence of individual studies on the overall SMD was shown. The middle vertical axis indicates the overall SMD and the two vertical axes indicate the 95%CI. Hollow circles represent the pooled SMD when the remaining study is omitted from the meta-analysis.

**
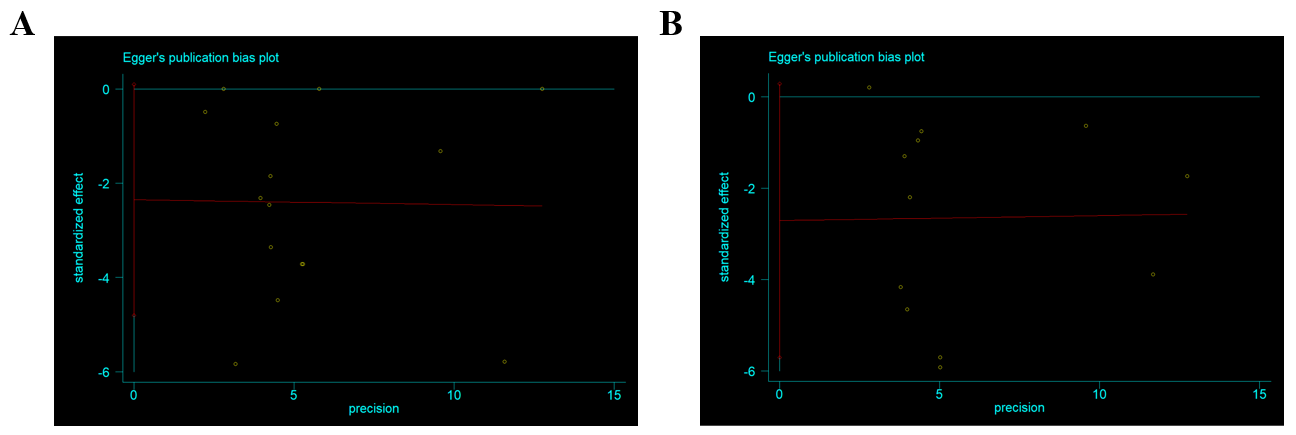
**

**Supplemental Figure 2 Funnel plot of detailing publication bias in the studies.** (A) blood *α*-carotene levels in patients with dementia; (B) blood lutein levels in patients with dementia.


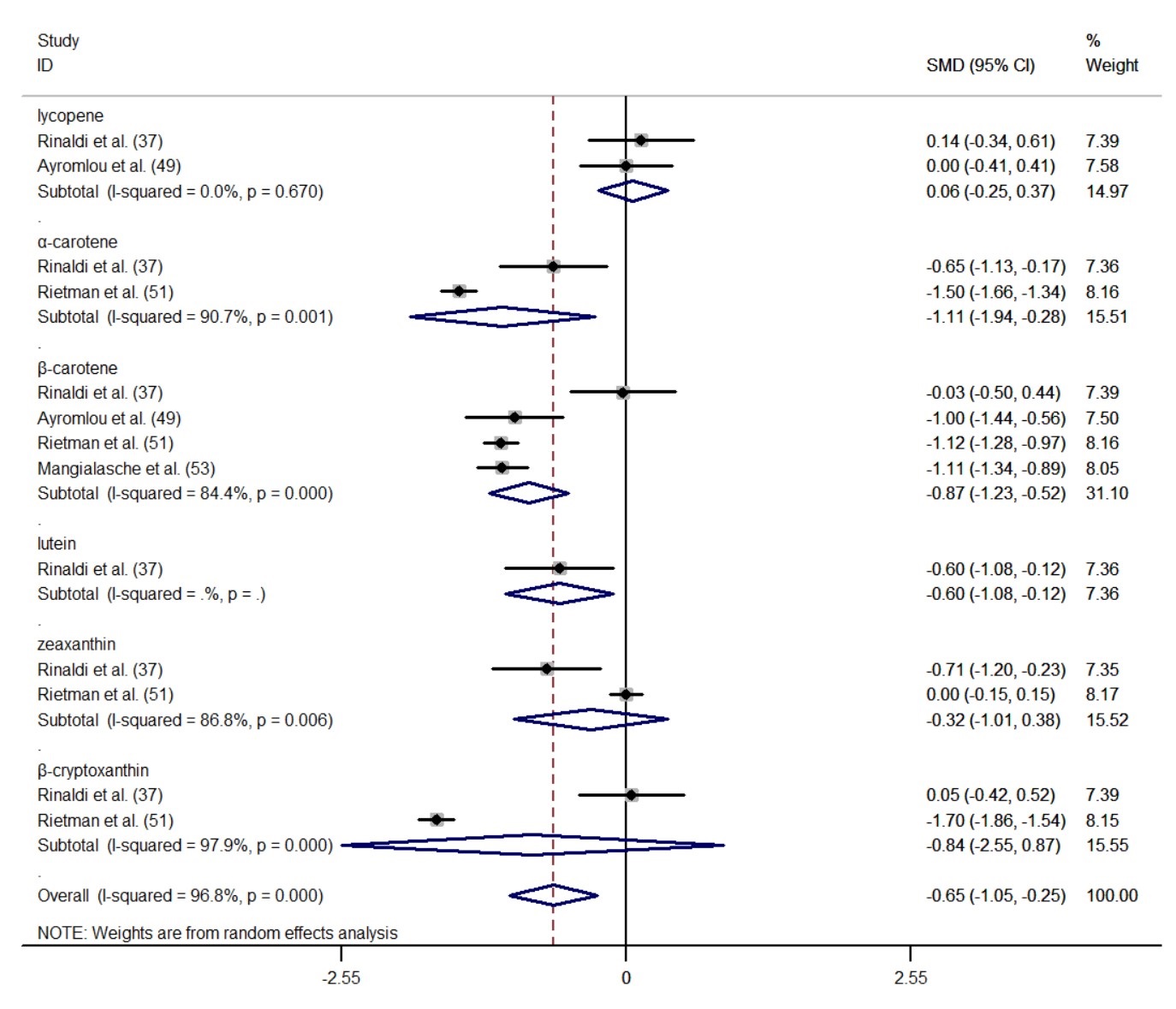


**Supplemental Figure 3 Forest plot of blood carotenoid levels between patients with MCI and HC subjects.** The data are expressed as SMDs with 95% CIs. The overall effect is represented by a hollow diamond. The horizontal lines represent 95% CI. The sizes of the shaded squares are proportional to study weight.


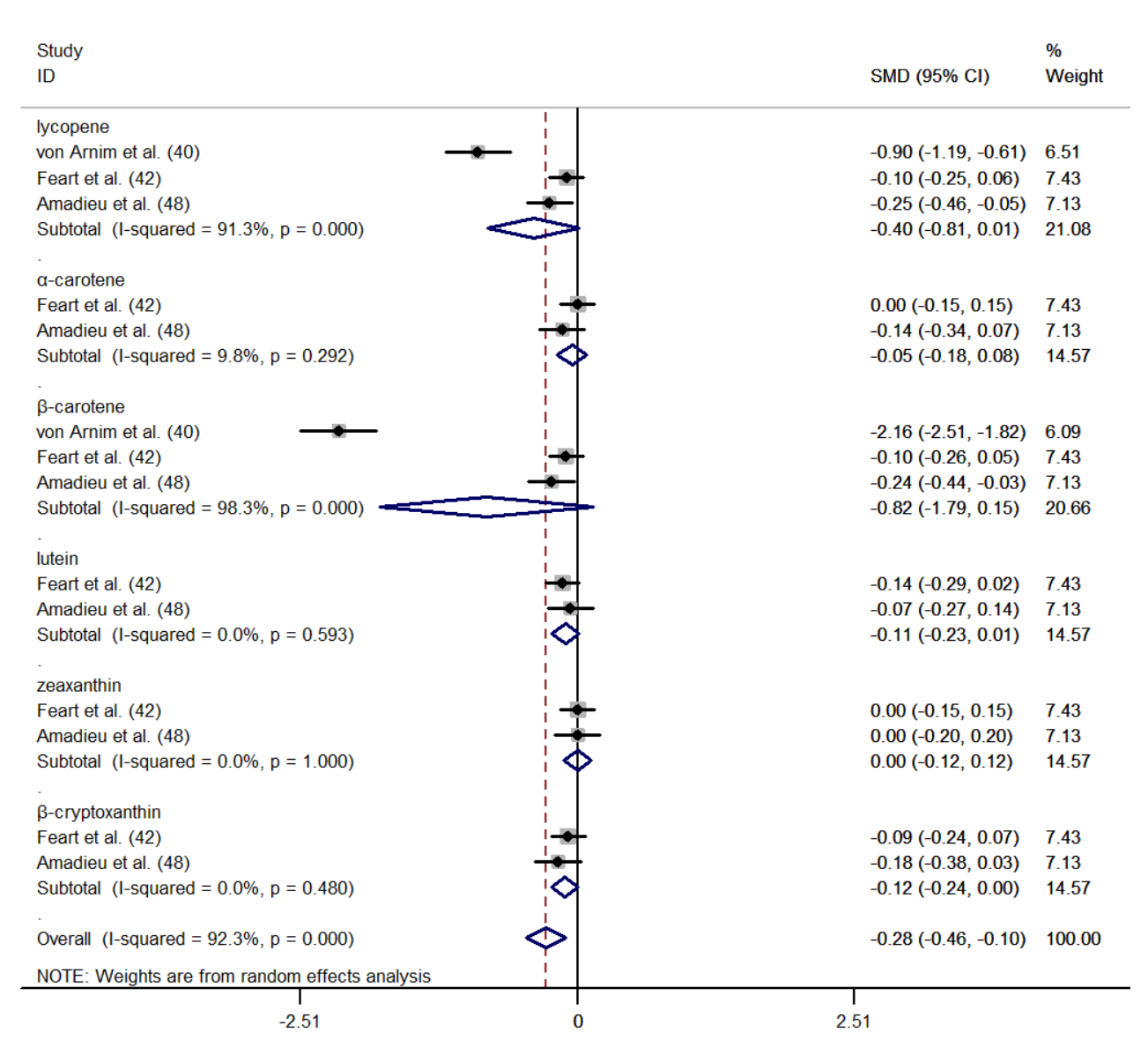


**Supplemental Figure 4 Forest plot of blood carotenoid levels between patients with indefinite dementia (ID) and HC subjects.** The data are expressed as SMDs with 95% CIs. The overall effect is represented by a hollow diamond. The horizontal lines represent 95% CI. The sizes of the shaded squares are proportional to study weight.
